# Metabolic Engineering of *Yarrowia lipolytica* for De Novo Biosynthesis of Umbelliferone

**DOI:** 10.3390/biom16101450

**Published:** 2026-10-04

**Authors:** Fengliu Lu, Ying Luo, Zhen Lei, Changfu Li, Yansheng Zhang

**Affiliations:** Shanghai Key Laboratory of Bio-Energy Crops, School of Life Sciences, Shanghai University, Shanghai 200444, China; lufengliu8857@shu.edu.cn (F.L.); luoy928@shu.edu.cn (Y.L.); 17379342467@shu.edu.cn (Z.L.)

**Keywords:** umbelliferone, *p*-coumaric acid, *Yarrowia lipolytica*, metabolic engineering

## Abstract

Umbelliferone, a key precursor for the biosynthesis of high-value furanocoumarin derivatives, is produced via the shikimate pathway. The oleaginous yeast *Yarrowia lipolytica* possesses an inherently robust shikimate pathway; however, de novo biosynthesis of umbelliferone in this microbial chassis has not been realized to date. Low intracellular pools of *p*-coumaric acid and suboptimal catalytic activity of downstream hydroxylation and cyclization enzymes represent the major bottlenecks limiting efficient umbelliferone production. In this study, we systematically screened five PAL/TAL variants, three 4CL isoforms, and two C2′H enzymes derived from diverse plant and microbial sources. The bifunctional enzyme RtPAL-TAL exhibited the highest productivity of *p*-coumaric acid, while the combination of At4CL3 and RgC2′H achieved the maximum umbelliferone accumulation. Expression of feedback-insensitive shikimate pathway mutant enzymes effectively relieved intrinsic pathway feedback inhibition, resulting in a 10.2-fold improvement in *p*-coumaric acid titer. All synthetic biosynthetic gene cassettes were stably integrated into the *Y*. *lipolytica* genome to generate the engineered strain YL-PU, which yielded 3.29 mg/L umbelliferone in shake-flask cultures. Further fed-batch fermentation in a 5 L bioreactor increased the umbelliferone titer to 125.52 mg/L at 84 h of cultivation. This work establishes the first de novo umbelliferone biosynthesis pathway in *Y. lipolytica and* identifies that C2′H represents a major candidate bottleneck under the tested configuration, in agreement with earlier findings. These findings provide a robust foundation for further metabolic engineering of the *Y*. *lipolytica* chassis to enable high-titer production of coumarins and other phenylpropanoid natural products.

## 1. Introduction

Umbelliferone (7-hydroxycoumarin) is a naturally occurring coumarin that exhibits extensive pharmacological activities, including antioxidant, anti-inflammatory, antimicrobial, antitumor, and neuro-protective effects [1]. Due to its versatile bioactivities, umbelliferone is widely applied in pharmaceuticals, cosmetics, food additives, and agricultural pesticides [2,3]. More importantly, umbelliferone serves as a central precursor for synthesizing many valuable furanocoumarins and prenylated coumarin derivatives, such as osthole, xanthotoxin, and bergapten [4,5]. These derivatives have gained increasing attention for the development of drugs and plant protection agents [6].

**Figure 1:**
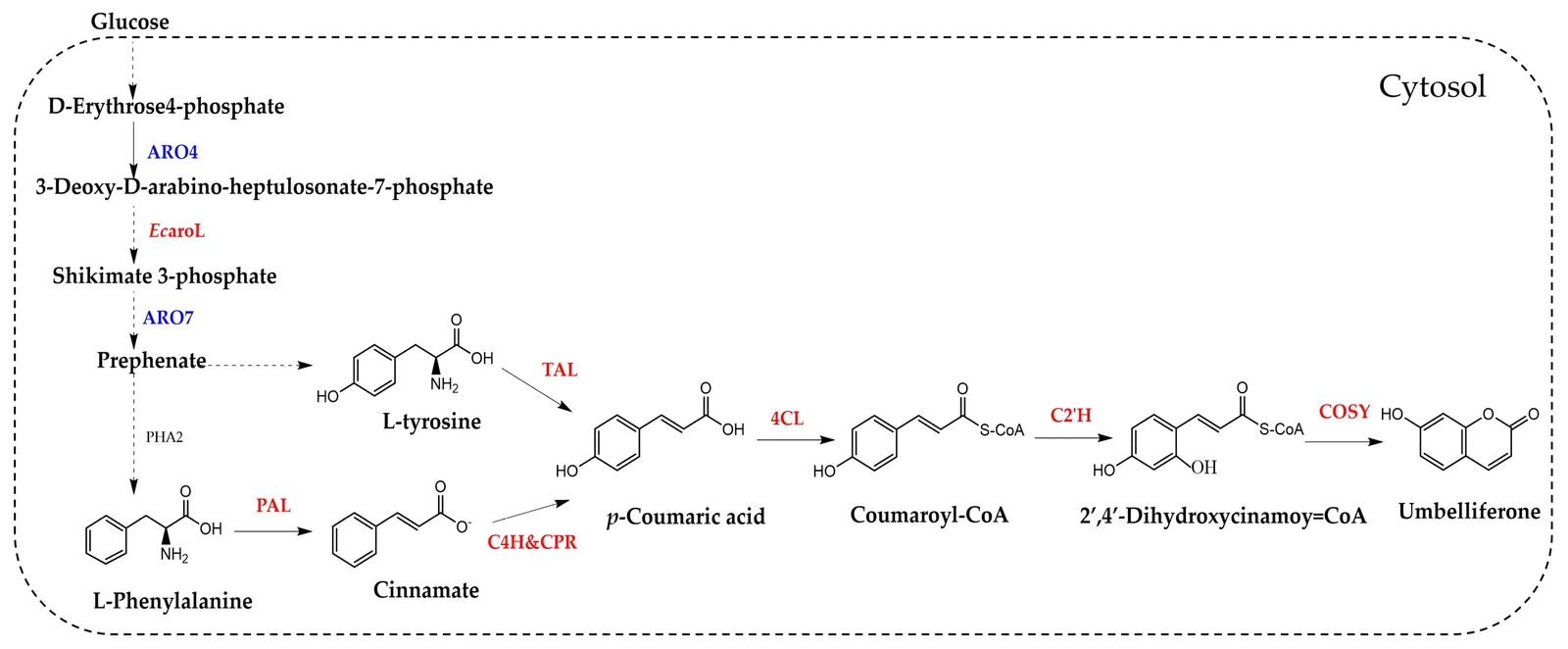
Proposed biosynthetic pathway for umbelliferone production in engineered yeast. ARO4, 3-deoxy-D-arabino-heptulosonate-7-phosphate synthase; ARO7, chorismate mutase; EcaroL, shikimate kinase; PHA2, prephenate dehydratase; PAL, phenylalanine ammonia-lyase; TAL, tyrosine ammonia-lyase; C4H, cinnamate-4-hydroxylase; CPR, cytochrome P450 reductase; 4CL, 4-coumarate-CoA ligase; C2′H, 4-coumaroyl-CoA 2′-hydroxylase; COSY, coumarin synthase. Genes labeled in red are artificially synthesized based on the codon usage bias of *Y. lipolytica*, while genes labeled in blue are native genes amplified from the genomes of *Y. lipolytica* or *S. cerevisiae*.

The biosynthesis of umbelliferone follows the phenylpropanoid pathway (Figure 1): tyrosine ammonia-lyase (TAL) catalyzes the conversion of L-tyrosine to *p*-coumaric acid [7], while phenylalanine ammonia-lyase (PAL) catalyzes the conversion of L-phenylalanine to cinnamic acid. The latter intermediate is then hydroxylated by cinnamate 4-hydroxylase (C4H) to yield *p*-coumaric acid [8]; coenzyme A is attached to *p*-coumaric acid by 4-coumarate-CoA ligase (4CL) [9], resulting in *p*-coumaroyl-CoA; subsequently, *p*-coumaroyl-CoA is hydroxylated at the C2′ position by *p*-coumaroyl-CoA 2′-hydroxylase (C2′H) [10]; and in a final step, the intermediate 2′,4′-dihydroxycinnamoyl-CoA undergoes isomerization and lactonization to form umbelliferone, which can be efficiently catalyzed by coumarin synthase (COSY) [11]. Among these steps, the selection of highly active enzymes, the supply of *p*-coumaric acid precursor, and the catalytic efficiency of downstream hydroxylation and cyclization are key bottlenecks restricting the de novo biosynthesis of umbelliferone [12].

Despite the wide spectrum of favorable pharmacological activities exhibited by umbelliferone, the extraction and purification of this compound and its derivatives from medicinal plants are severely restricted by their low in-situ content and inherent seasonal and geographical constraints [13]. On the other hand, chemical synthesis involves harsh reaction conditions, toxic catalysts, complicated byproducts, and severe environmental pollution [14,15,16], which are incompatible with the requirements of green and sustainable manufacturing. Microbial biosynthesis of umbelliferone via heterologous reconstitution of plant biosynthetic pathways has been demonstrated in *Escherichia coli* [17,18]. Nevertheless, its de novo production titer in *E. coli* remains relatively low, reaching below 3.0 mg/L [18]. By contrast, Yang and coworkers reported de novo synthesis of umbelliferone at 66.1 mg/L using glucose-fed engineered *E. coli* [17]. Supplementation with exogenous *p*-coumaric acid further enabled the titer to reach 82.9 mg/L in this chassis [17]. Very recently, fed-batch fermentation of engineered *Saccharomyces cerevisiae* achieved umbelliferone biosynthesis with a titer of 82.04 mg/L [4].

The oleaginous yeast *Yarrowia lipolytica* possesses an inherently robust shikimate pathway, which efficiently supplies aromatic amino acids including L-tyrosine and L-phenylalanine—the direct precursors for umbelliferone biosynthesis [19]. To date, however, there have been no reports regarding the engineering of *Y. lipolytica* for umbelliferone synthesis. Herein, we evaluated the potential of *Y. lipolytica* to produce umbelliferone.

## 2. Materials and Methods

### 2.1. Strains and Reagents

All *Yarrowia lipolytica* strains used in this study were derived from the chassis strain ‘SC52 + SC59’ (MatA, leu2-270, ura3-302, xpr2-322, axp2-delta NU49, XPR2::SUC2, AXP::ScRAD52 + ScRAD59), which exhibits significantly improved homologous recombination efficiency [20]. *E. coli* DH5α was used for plasmid amplification and construction.

Yeast strains were routinely cultivated at 30 °C. YPD medium (10 g/L yeast extract, 20 g/L peptone, 20 g/L glucose) was used for strain propagation and fermentation. Synthetic dropout (SD) medium (6.7 g/L yeast nitrogen base without amino acids, 20 g/L glucose, supplemented with appropriate amino acids) was used for screening transformants. Solid media were prepared by adding 20 g/L agar. *E. coli* strains were grown at 37 °C in Luria–Bertani (LB) medium (10 g/L tryptone, 5 g/L yeast extract, 10 g/L NaCl) with 100 mg/L ampicillin for plasmid maintenance.

The *p*-coumaric acid and umbelliferone standards were purchased from Sigma-Aldrich (St. Louis, MO, USA). Restriction enzymes, DNA ligase, and Taq DNA polymerase were purchased from Thermo Fisher Scientific (Waltham, MA, USA). All heterologous genes were codon-optimized for *Y. lipolytica* and synthesized commercially, and their cDNA sequences are shown in Table S1.

### 2.2. Construction of Yeast Expression Vectors

Details regarding the construction of yeast expression vectors are provided in the Supplemental Materials. Briefly, plasmids were built using the backbones of pHR_AXP_hrGFP [21] or pCAS1yl [22]. The plasmids made by this study are shown in Table S2, and the primers used in this study are presented in Table S3.

### 2.3. Screening of High-Activity Biosynthetic Enzymes

According to the biosynthetic pathway of *p*-coumaric acid and umbelliferone, five enzymes with potential catalytic functions were selected from different sources for activity screening, including phenylalanine ammonia-lyase (PAL)/tyrosine ammonia-lyase (TAL), 4-coumarate-CoA ligase (4CL) and *p*-coumaroyl-CoA 2′-hydroxylase (C2′H). The screened PAL/TAL genes included FjTAL from *Flavobacterium johnsoniae* [23], ClTAL from *Chryseobacterium luteum* [24], AtPAL from *Arabidopsis thaliana* [25], SmPAL from *Streptomyces maritimus* [26], and the bifunctional RtPAL-TAL from *Rhodotorula toruloides* [27]. The 4CL genes contained At4CL3 from *A. thaliana* [28], Sc4CL from *Streptomyces coelicolor* [29] and Gm4CL4 from *Glycine max* [30]. The C2′H genes were AdC2′H derived from *Angelica decursiva* and RgC2′H derived from *Rhodotorula glutinis* [31]. For C4H and CPR, the *A. thaliana*-derived AtC4H and AtCPR were employed. All the candidate genes were codon-optimized according to the codon preference of *Y. lipolytica* and chemically synthesized.

The target genes were individually inserted downstream of either the pTEFin or pUAS1B8TEF promoter. These promoters were carried on yeast episomal vectors pHRm-URA3 and pYarrow-LEU2, which were derived from pHR_AXP_hrGFP and pCAS1yl, respectively. These two episomal plasmids were constructed in the present study, and comprehensive construction details are available in the supplementary file. The recombinant plasmids were individually or co-transformed into the chassis strain *Y. lipolytica* ‘Sc52 + SC59’ using the Frozen-EZ Yeast Transformation II Kit (Zymo Research, CA, USA). After screening on SD selective plates, positive recombinant strains were obtained and subjected to shake-flask fermentation for enzyme activity evaluation. To screen PAL/TAL enzymes with high activity, fermentation liquor was sampled for HPLC analysis at 303 nm. Variations in *p*-coumaric acid biosynthesis directly reflected the catalytic efficiency of distinct PAL/TAL enzymes in *Y. lipolytica*. For the combinatorial screening of 4CL and C2′H, different gene pairs were co-expressed in the *p*-coumaric acid-producing strain, and umbelliferone production was measured by HPLC at 322 nm to assess catalytic performance.

### 2.4. Genomic Integration of the Complete Umbelliferone Biosynthetic Pathway in Yeast

CRISPR–Cas9 combined with homologous recombination was applied for site-specific gene integration in *Y. lipolytica*. For the construction of *p*-coumaric acid producer, the MFE1 locus-specific plasmid harboring *AtC4H*, *AtCPR*, *RtPAL-TAL*, *ScARO4^K229L^*, *ScARO7^G141S^* and *EcaroL* was transformed into ‘SC52 + SC59’ with the Frozen-EZ Yeast Transformation II Kit (Zymo Research). Transformants were initially screened on URA3-deficient SD plates and validated by colony PCR. Positive strains were then cultured on 5-FOA plates to cure free plasmids and eliminate the URA3 marker, yielding marker-free host strains for re-transformation.

Subsequently, the umbelliferone biosynthetic genes *AtCOSY*, *RgC2′H* and *At4CL3* were integrated at the D17 locus based on the *p*-coumaric acid-producing strain. The same transformation and screening cycles were performed iteratively. All target genes were finally integrated into the yeast genome. The resultant strain YL-PU could stably produce umbelliferone. A summary of all integrated strains constructed in this study is provided in Table S4.

### 2.5. Cultivation of the Engineered Strains

For shake-flask fermentation, 2 μL of glycerol stock of engineered yeast preserved at −80 °C was streaked onto YPD solid plates, which were then incubated upside down at 30 °C for 2–3 days. A single colony was picked and inoculated into 3 mL of fresh YPD liquid medium, followed by cultivation at 30 °C and 220 rpm for 1–2 days until the cells reached the mid-log phase (OD_600_ = 0.6~0.8). Subsequently, the seed culture was transferred into conical flasks containing 20 mL of YPD liquid medium with an inoculum size of 10–20%. The fermentation was carried out at 30 °C and 220 rpm for 5 days.

Fed-batch fermentation was performed in a 5 L fermentor (Beijing Mancang Equipment Co. Ltd., Beijing, China). The basal medium (per L) contained 40 g glucose, 7.5 g (NH_4_)_2_SO_4_, 14.4 g KH_2_PO_4_, 0.5 g MgSO_4_·7H_2_O, 2 mL trace element stock solution (FeSO_4_·7H_2_O, 2.8 g/L; ZnSO_4_·7H_2_O, 5.75 g/L; CaCl_2_·2H_2_O, 2.9 g/L; MnCl_2_·4H_2_O, 0.32 g/L; CoCl_2_·6H_2_O, 0.47 g/L; CuSO_4_·5H_2_O, 0.5 g/L; Na_2_MoO_4_·2H_2_O, 0.48 g/L; EDTA-Na, 40 mM), and 1 mL vitamin stock solution (D-biotin, 0.05 g/L; D-calcium pantothenate, 1.0 g/L; nicotinic acid, 1.0 g/L; 4-aminobenzoic acid, 0.2 g/L; myo-inositol, 25 g/L; thiamine hydrochloride, 1.0 g/L). Single colonies of the engineered *Y. lipolytica* YL-PU strain were inoculated into 10 mL YPD and cultured at 30 °C for 24 h as primary seeds. The culture was sub-cultured in 250 mL YPD and incubated at 30 °C for 24 h to prepare secondary seeds. A total of 250 mL of the secondary seed culture was inoculated into 2.5 L of basal medium in the fermentor. Fermentation was conducted at 28 °C, with 3.0 vvm aeration and agitation controlled at 300–900 rpm to maintain dissolved oxygen ≥ 20%. The pH was kept at 5.5 using 5 M NH_4_OH. After initial glucose depletion, fed-batch feeding began with 600 g/L glucose solution. The feeding strategy was programmed as follows: no feeding from 0 to 56 h; 5.3 mL/h from 56 to 71 h; 14.5 mL/h from 71 to 80 h; and 20.4 mL/h from 80 to 96 h, to keep the residual glucose concentration below 1 g/L. Sampling was performed every 12 h. A 10 mL aliquot of fermentation broth was withdrawn to measure the OD_600_ value and quantify the titers of *p*-coumaric acid and umbelliferone.

### 2.6. Metabolite Extraction and Analysis

Fermentation broth was centrifuged at 4000 rpm for 8 min to separate the supernatant and cell pellets. For shake-flask cultures, the extracellular fraction was obtained by extracting 1.0 mL of fermentation supernatant with an equal volume of ethyl acetate. The ethyl acetate extract was evaporated to dryness and reconstituted in 100 μL methanol for HPLC analysis. For the intracellular fraction, yeast cells were harvested from 5 mL fermentation broth by centrifugation. Approximately 500 μL glass beads and 500 μL sodium phosphate buffer (pH 7.0) were added for cell lysis. After lysis, 1 mL ethyl acetate was added to the cell lysate, followed by vortexing for 5 min. The mixture was centrifuged at 12,000 rpm for 5 min, and the upper organic phase was collected. This extraction procedure was repeated three times. Combined organic phases were transferred to a 2 mL EP tube, and ethyl acetate was removed by evaporation. The residue was re-dissolved in 100 μL methanol and centrifuged at 12,000 rpm for 3 min, and the resulting supernatant was subjected to HPLC analysis.

For fed-batch fermentation cultures, the extracellular fraction was obtained by mixing 100 μL of fermentation broth with an equal volume of methanol in a 1.5 mL microcentrifuge tube, followed by votexing for 2–3 min. The mixture was centrifuged at 12,000 rpm for 3 min, and the resultant supernatant was directly analyzed by HPLC. The intracellular fraction was extracted using the same protocol described for shake-flask cultures.

HPLC-DAD analysis was carried out using a Shimadzu CBM-20A system (Shimadzu, Kyoto, Japan) coupled with a diode-array detector (DAD). Separation was performed on a ZORBAX SB-C18 column (4.6 mm × 250 mm, 5 μm). The flow rate was set at 1.0 mL/min, and the injection volume was 10 μL. For *p*-coumaric acid, mobile phase A was 0.1% (*v*/*v*) formic acid in ultrapure water, and mobile phase B was acetonitrile. The column temperature was maintained at 30 °C, and the detection wavelength was 303 nm. The gradient elution program was as follows: 0.00 min, 5% B; 0.00–3.00 min, 5–10% B; 3.00–5.00 min, 10–30% B; 5.00–10.00 min, 30–70% B; 10.00–10.50 min, 70–90% B; 10.50–12.50 min, 90% B; 12.50–13.00 min, 90–5% B; 13.00–16.00 min, 5% B. For umbelliferone, ultrapure water served as mobile phase A and acetonitrile as mobile phase B. The column temperature was maintained at 35 °C, with a detection wavelength of 322 nm. The gradient elution program was set as follows: 0.01 min, 15% B; 0.01–20.00 min, 15–70% B; 20.00–40.00 min, 70% B; 40.00–40.01 min, 70–15% B; 40.01–45.00 min, 15% B; stop run at 45.01 min.

Quantification was performed via the external standard method with calibration curves (Figure S1). For both *p*-coumaric acid and umbelliferone, the tested concentration range of 0–200 µg/mL lies within the linear region with satisfactory linearity (Figure S1). The regression equation for *p*-coumaric acid was y = 21,879*x* − 7029.2 (*R^2^* = 0.9932), while that for umbelliferone was y = 38,590*x* + 80,003 (*R^2^* = 0.9993). Unless otherwise specified, the product concentrations presented in the text and figures represent the sum of intracellular and extracellular titers.

### 2.7. Statistical Analysis

All experiments were performed in at least three biological replicates, and the data were expressed as mean ± standard deviation (SD). Student’s *t* test was used for pairwise comparison, and one-way ANOVA with Tukey’s post hoc test was used for multiple comparisons, with *p* < 0.05 considered significant.

## 3. Results

### 3.1. Construction of the p-Coumaric Acid-Producing Yeast Strain

**Figure 2:**
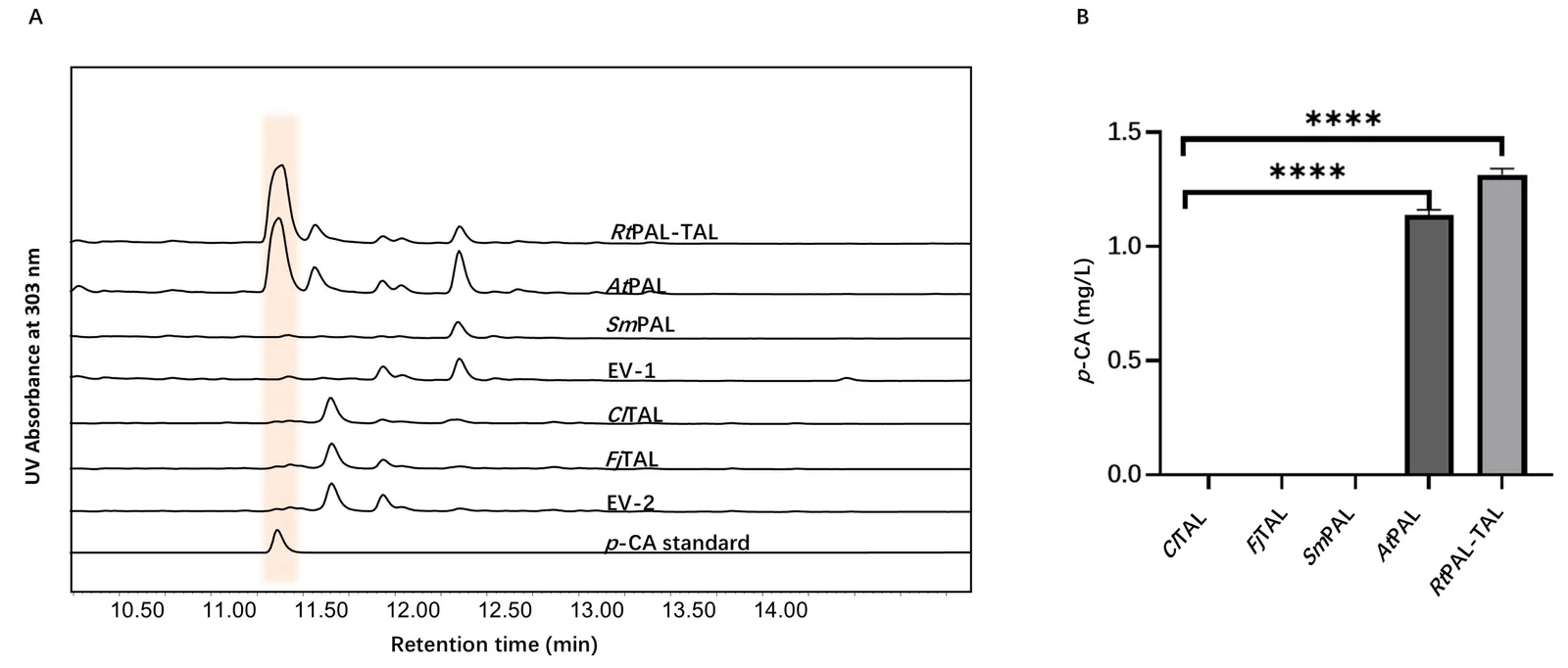
Screening of PAL/TAL enzymes for *p*-coumaric acid (*p*-CA) production in yeast. (**A**) HPLC chromatograms of culture extracts from the strains expressing different PAL- or TAL-encoding genes. The peak region corresponding to authentic *p*-CA standard is highlighted by an orange shaded area. EV-1 and EV-2 represent empty-vector control strains. (**B**) Quantitative determination of extracellular *p*-CA titers (mg/L). Data are presented as mean ± standard deviation (SD), with *n* = 3 biological replicates. Differences were considered statistically significant at *p* < 0.05. Significance levels were denoted as **** *p* < 0.0001.

We first constructed and systematically optimized the *p*-coumaric acid biosynthetic pathway by screening five PAL/TAL homologs of distinct phylogenetic origins. Each candidate enzyme was co-expressed with the *A. thaliana*-derived AtC4H and AtCPR in *Y. lipolytica*. Relative to the control strains (EV-1 and EV-2), strains expressing RtPAL-TAL and AtPAL produced a new chromatographic peak that matched the *p*-coumaric acid standard in both retention time (Figure 2A) and UV spectrum (Figure S2), with yields of 1.31 mg/L and 1.14 mg/L, respectively (Figure 2B). Strains heterologously expressing either FjTAL, SmPAL or ClTAL produced no detectable *p*-coumaric acid (Figure 2), even upon exogenous supplementation with 10 mM tyrosine (Figure S3).

**Figure 3:**
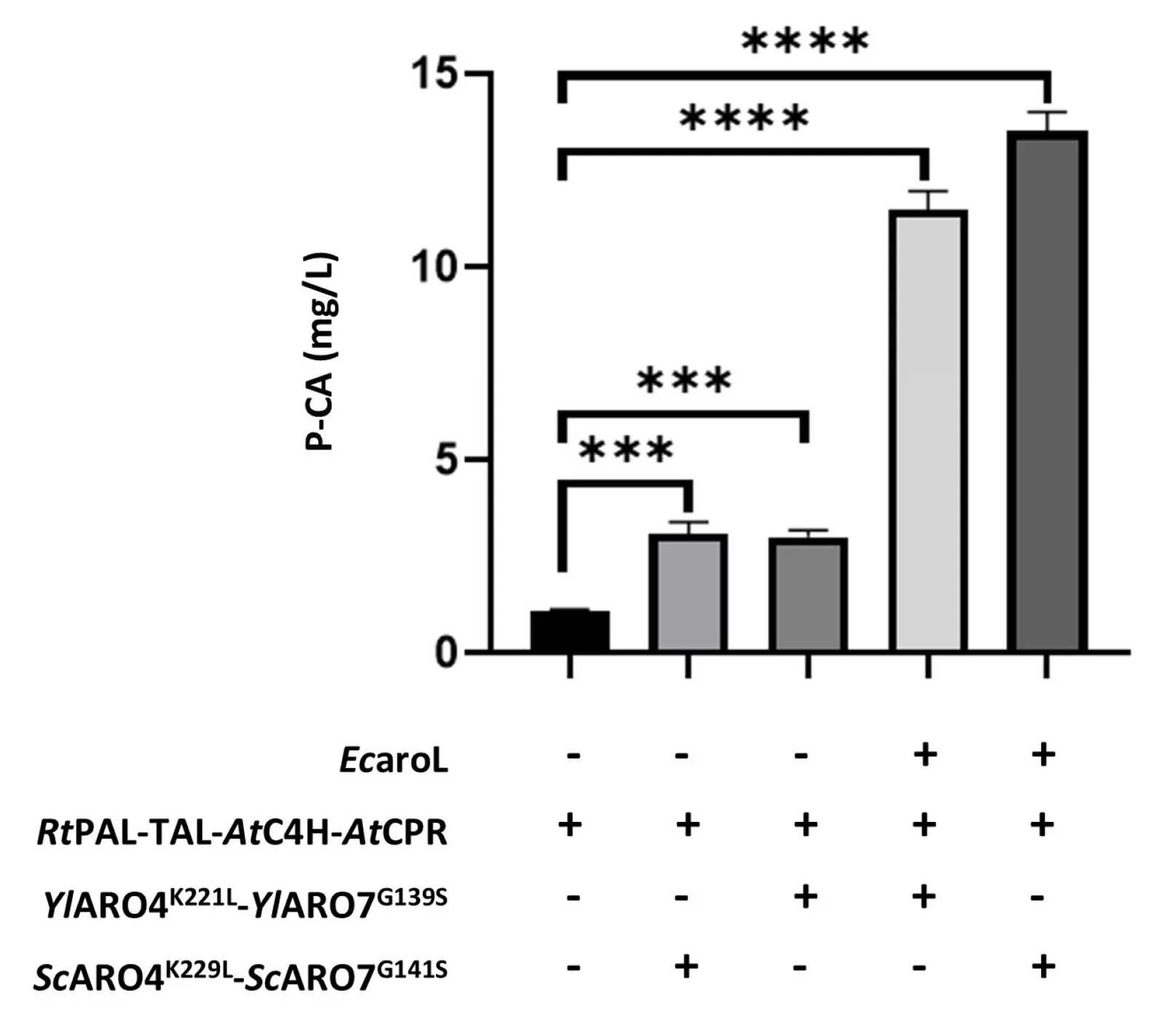
The effect of expressing the shikimate pathway genes on the production of *p*-coumaric acid. Data are presented as mean ± standard deviation (SD), with *n* = 3 biological replicates. Differences were considered statistically significant at *p* < 0.05. Significance levels were denoted as *** *p* < 0.001 and **** *p* < 0.0001.

To further expand the intracellular reservoir of aromatic amino acid precursors and relieve native feedback inhibition within the shikimate pathway, two sets of feedback-deregulated DAHP synthase and chorismate mutase mutant cassettes were constructed and comparatively characterized. The two modules were respectively sourced from *S. cerevisiae* (ScARO4^K229L^ and ScARO7^G141S^) and *Y. lipolytica* (YlARO4^K221L^ and YlARO7^G139S^). The shikimate kinase EcaroL from *E. coli* was co-expressed with each mutant module to strengthen the upstream shikimate metabolic flux. The strain harboring ScARO4^K229L^ and ScARO7^G141S^ produced *p*-coumaric acid at a titer of 13.31 mg/L, whereas the strain carrying YlARO4^K221L^ and YlARO7^G139S^ reached 12.88 mg/L (Figure 3). This demonstrated that the desensitized mutant enzymes derived from *S. cerevisiae* exhibited superior performance in boosting *p*-coumaric acid biosynthesis. The optimized strain containing the full pathway cascade delivered a 10.2-fold increase in *p*-coumaric acid titer relative to the chassis strain merely expressing RtPAL-TAL (Figure 3). Subsequently, the gene expression cassette carrying *RtPAL-TAL*, *AtC4H*, *AtCPR*, *ScARO4^K229L^*, *ScARO7^G141S^*, and *EcaroL* was integrated into the MFE1 locus of strain ‘Sc52 + Sc59’, generating the *p*-coumaric acid producer YL-P. Notably, HPLC analysis confirmed that *p*-coumaric acid was fully excreted into the culture supernatant, with no detectable intracellular product accumulation (Figure S4).

### 3.2. Construction of the Engineered Strain for Synthesis of Umbelliferone

**Figure 4:**
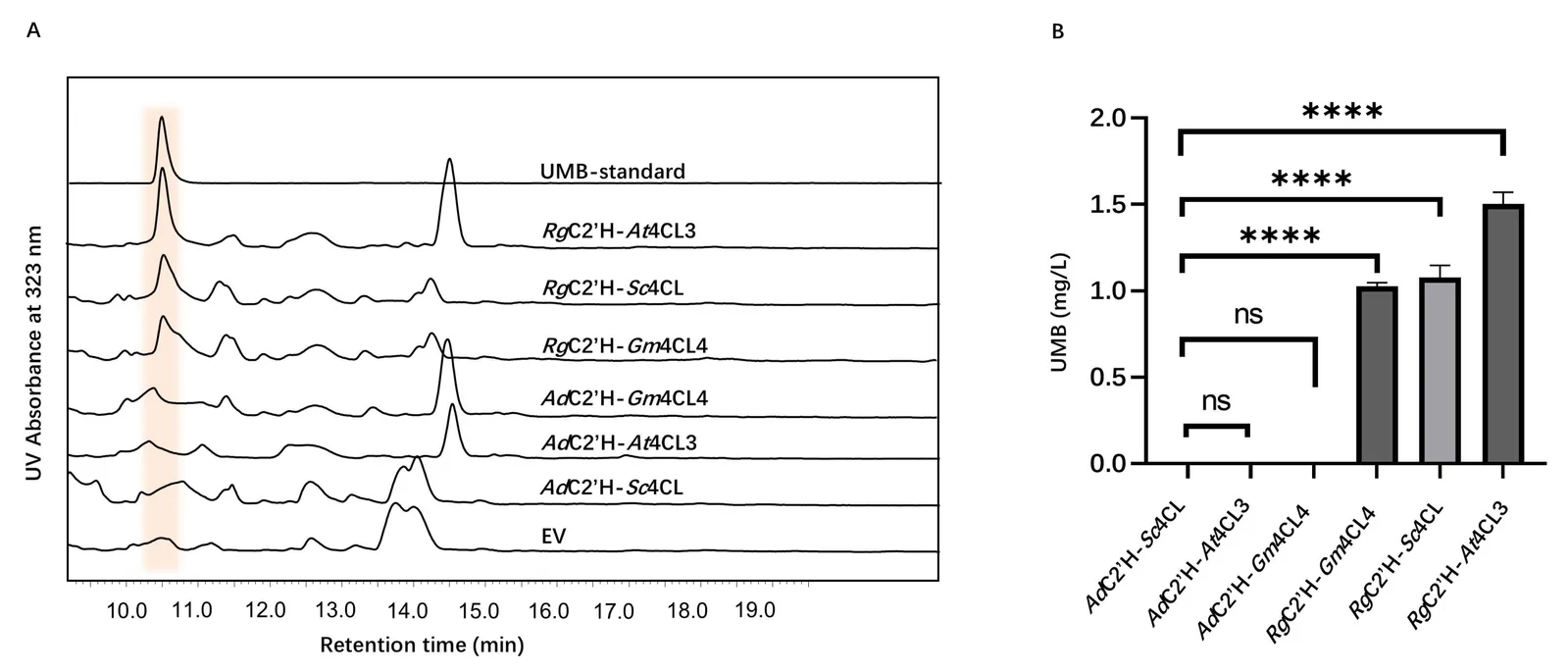
Combinatorial screening of C2′H and 4CL enzyme pairs for umbelliferone (UMB) biosynthesis. (**A**) HPLC profiles of fermentation samples. The retention-time region matching the umbelliferone (UMB) standard is highlighted by an orange shaded area; empty vector (EV) served as the negative control. (**B**) Quantified umbelliferone yields. Data are presented as mean ± standard deviation (SD), with *n* = 3 biological replicates. Differences were considered statistically significant at *p* < 0.05. Significance levels were denoted as **** *p* < 0.0001. ns, not significant.

Starting from the *p*-coumaric acid-producing chassis strain YL-P, we performed sequential screening of rate-limiting enzymes involved in umbelliferone biosynthesis—specifically 4-coumarate-CoA ligase (4CL) and *p*-coumaroyl-CoA 2′-hydroxylase (C2′H)—to identify optimal enzyme combinations. Three 4CL variants (At4CL3 from *A. thaliana*, Sc4CL from *S. coelicolor*, and Gm4CL4 from *G. max*) were pairwise co-expressed with two C2′H orthologs (AdC2′H from *A. decursiva* and RgC2′H from *R. glutinis*) in an intermediate strain pre-integrated with AtCOSY, the cyclase gene responsible for intramolecular lactonization. The strains bearing AdC2′H failed to synthesize measurable umbelliferone, while all RgC2′H-expressing recombinants accumulated target products. The combination of At4CL3 and RgC2′H delivered the maximum umbelliferone production among all tested pairs (Figure 4).

**Figure 5:**
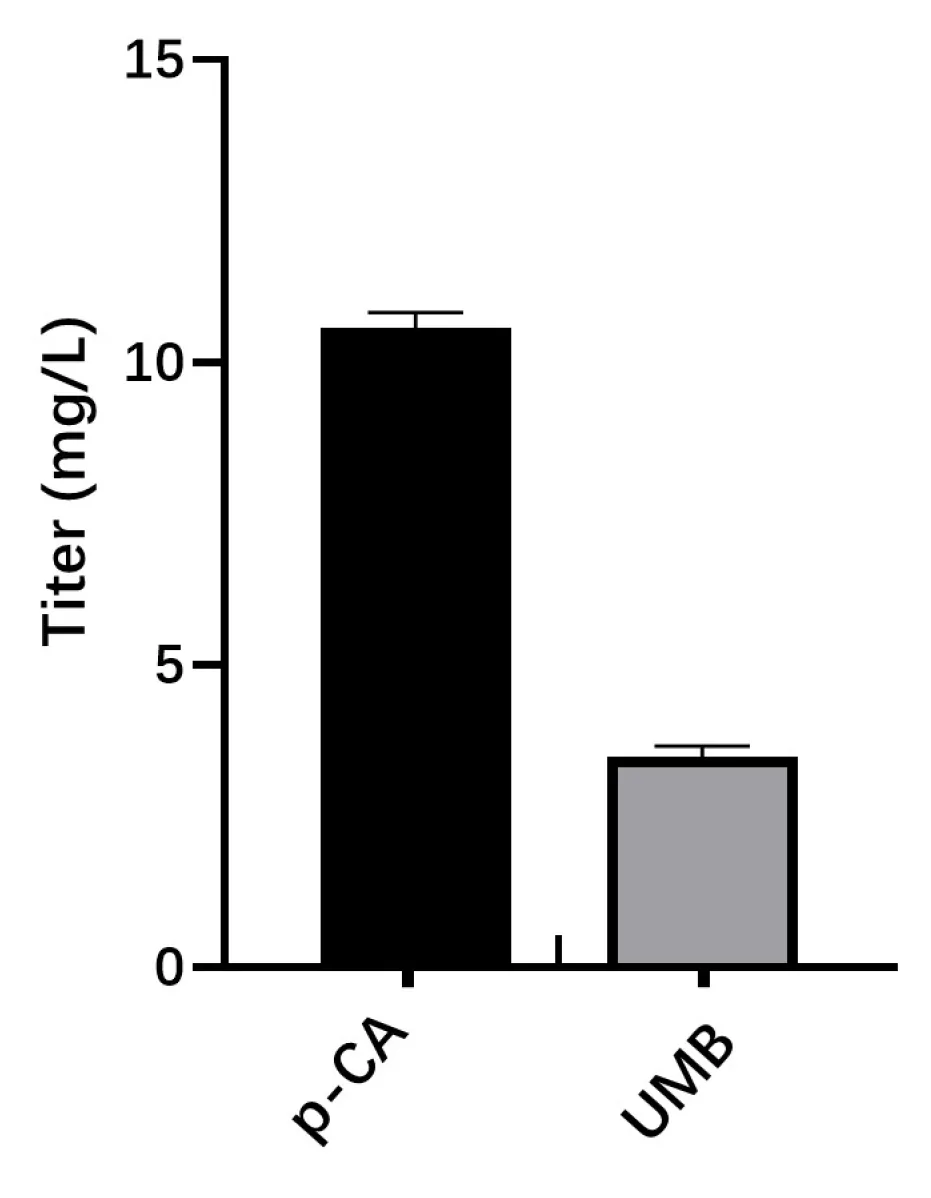
Comparison of extracellular *p*-coumaric acid (p-CA) and umbelliferone (UMB) titers in the engineered yeast strain YL-PU under shake-flask fermentation. Data are presented as mean ± standard deviation (SD), with *n* = 3 biological replicates.

The optimized gene cassette containing *At4CL3*, *RgC2′H* and *AtCOSY* was chromosomally integrated into YL-P to generate the stable umbelliferone producer YL-PU. Most of umbelliferone synthesized by YL-PU was secreted extracellularly, with only trace amounts retained intracellularly (Figure S5). The resulting strain YL-PU produced 3.39 ± 0.15 mg/L umbelliferone and 10.33 ± 0.51 mg/L *p*-coumaric acid in shake flask cultivation (Figure 5).

### 3.3. Fermentation Performance of the Strain YL-PU Under Fed-Batch Cultivation

**Figure 6:**
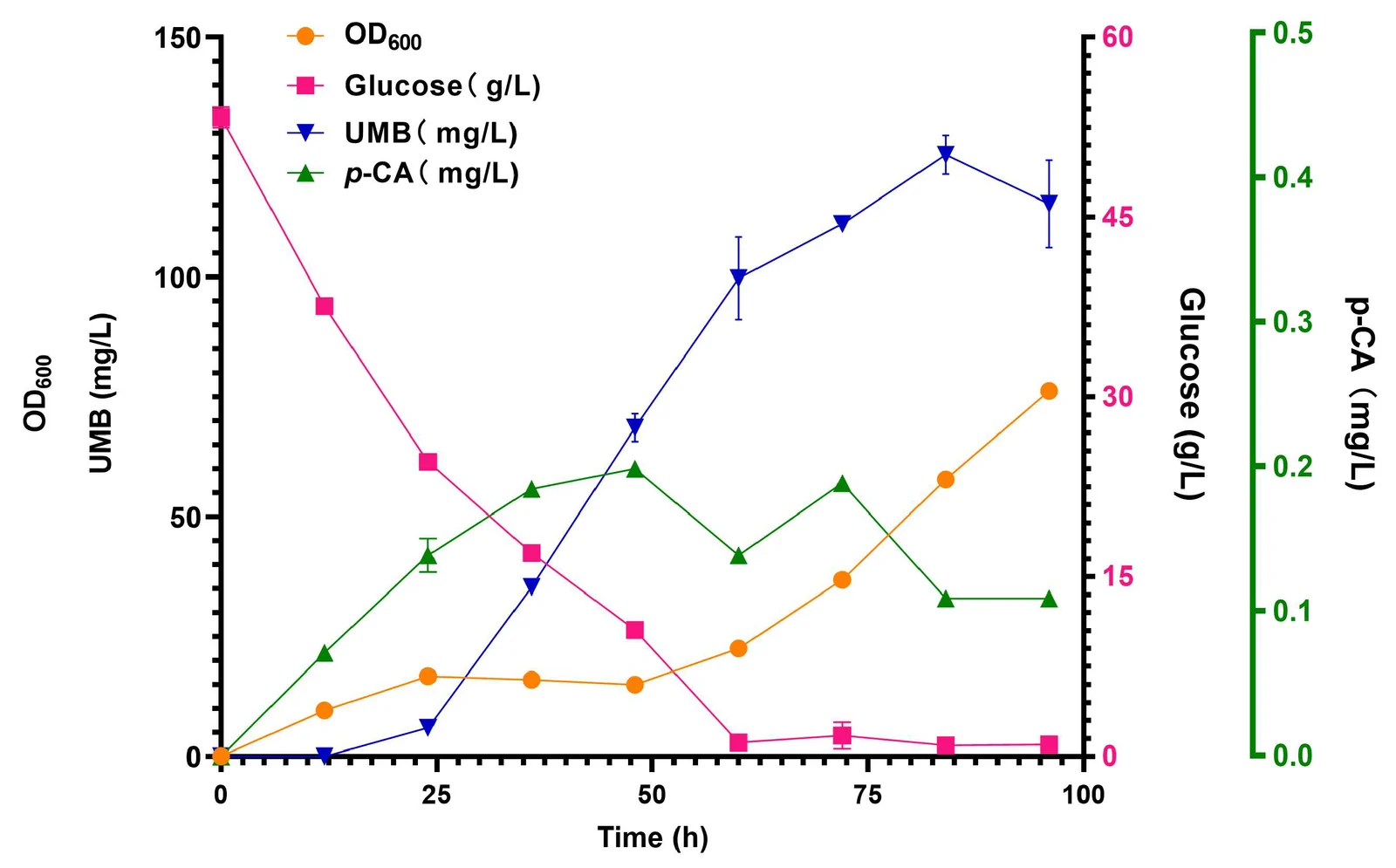
Fermentation performance of engineered strain YL-PU in a 5-L fed-batch bioreactor. Time-course profiles of cell growth (OD_600_), residual glucose consumption, intermediate *p*-coumaric acid (p-CA) accumulation and umbelliferone (UMB) production were monitored over 96 h. The left vertical axis corresponds to OD_600_ and UMB concentration (mg/L); the right vertical axis denotes glucose (g/L) and *p*-CA (mg/L). Bioreactor cultivations were performed in duplicates. Data are presented as averages from both reactors, and error bars represent standard deviation.

To evaluate the industrial application potential of the engineered strain YL-PU, fed-batch fermentation was conducted in a 5 L bioreactor with glucose as the sole carbon source. The cell density (OD_600_) increased rapidly and reached 79.13 at 96 h of cultivation. The umbelliferone titer peaked at 125.52 mg/L at 84 h, when the OD_600_ was 57.93, and subsequently decreased to 115.24 mg/L at 96 h (Figure 6). Interestingly, the concentration of *p*-coumaric acid was maintained at only 0.1–0.2 mg/L throughout the fermentation.

## 4. Discussion

Despite the fact that *Y. lipolytica* naturally harbors a strong shikimate pathway [19], which efficiently furnishes L-tyrosine and L-phenylalanine as direct precursors of umbelliferone, the de novo production of umbelliferone in *Y. lipolytica* has not been documented to date. Herein, we report the first de novo biosynthesis of umbelliferone engineered in *Y. lipolytica*. In shake-flask cultures without any metabolic pathway optimization, the engineered strain produced 3.39 mg/L umbelliferone. This titer is comparable to the de novo umbelliferone titer of 2.43 mg/L obtained via shake-flask cultivation in *E. coli*, as reported by Yan et al. [18], yet considerably lower than the 73.05 mg/L achieved in *S. cerevisiae* [4]. Such discrepancies can be largely attributed to the adoption of heterologous enzymes derived from different sources.

*p*-Coumaric acid serves as the key precursor of umbelliferone. To optimize its biosynthesis, five enzymes were screened and characterized for their catalytic performance in producing *p*-coumaric acid, including two phenylalanine ammonia-lyases (AtPAL and SmPAL), two tyrosine ammonia-lyases (FjTAL and ClTAL), and one bifunctional PAL/TAL enzyme (RtPAL-TAL). A previous study has demonstrated that SmPAL exhibits over 20-fold higher catalytic activity than AtPAL [32]. Nevertheless, heterologous expression of SmPAL in *Y. lipolytica* failed to yield detectable *p*-coumaric acid, whereas functional AtPAL expression successfully drove the biosynthesis of this product (Figure 2). In addition, neither of the two tested TALs supported *p*-coumaric acid synthesis in the engineered yeast strain (Figure 2), despite a prior report verifying the robust catalytic activity of FjTAL in *Y. lipolytica* [33]. The underlying mechanism for the functional failure of these enzymes remains unclear. We hypothesize that SmPAL, FjTAL, and ClTAL may be expressed as insoluble aggregates in our modified yeast strain, thereby completely losing their catalytic activities. Notably, the bifunctional enzyme RtPAL-TAL from *R. toruloides* displayed superior catalytic performance to AtPAL in *p*-coumaric acid production (Figure 2). This advantage is likely attributed to its dual substrate affinity for both L-phenylalanine and L-tyrosine. This finding is consistent with the previous conclusion that bifunctional PAL-TAL enzymes are more efficient than monofunctional homologs in constructing aromatic carbon skeletons for downstream biosynthesis [34]. Precursor limitation is widely acknowledged as the predominant bottleneck restricting microbial *p*-coumaric acid biosynthesis. This limitation arises from stringent feedback inhibition targeting two key regulatory nodes of the shikimate pathway: DAHP synthase (ARO4) and chorismate mutase (ARO7) [35]. To address this constraint, we co-expressed two feedback-insensitive mutant enzymes derived from *S. cerevisiae* (ScARO4^k229L^ and ScARO7^G141S^), along with the heterologous shikimate kinase EcaroL from *E. coli*. This combinatorial engineering strategy successfully alleviated dual-site feedback inhibition and augmented the rate-limiting shikimate phosphorylation step, consistent with the shikimate pathway engineering strategy reported by Gu et al. [19]. As a result, the *p*-coumaric acid titer was elevated 10.2-fold, reaching 13.31 mg/L (Figure 3).

The bioconversion of *p*-coumaric acid to umbelliferone is catalyzed sequentially by three enzymes, namely 4CL, C2′H, and AtCOSY (Figure 1). To optimize the umbelliferone biosynthetic pathway in *Y. lipolytica*, we systematically combined and characterized three 4CL isoforms (Gm4CL4, Sc4CL, and At4CL3) and two C2′H variants (RgC2′H and AdC2′H) for their catalytic performance. Significant umbelliferone accumulation was detected exclusively in strains harboring RgC2′H, while no product was observed in those expressing AdC2′H, indicating that AdC2′H from *A. decursiva* is inactive in *Y. lipolytica*. Interestingly, a previous study demonstrated that plant-derived AdC2′H exerts catalytic activity in *S. cerevisiae*, whereas fungal RgC2′H fails to function in this conventional yeast host [4]. The molecular mechanism underlying this host-dependent functional reversal of the two C2′H enzymes remains elusive, which we hypothesize is attributed to the distinct intracellular microenvironments of the two yeast species. Previous heterologous expression assays in *E. coli* have ranked the catalytic activity of 4CL isoforms in the order of Gm4CL4 > Sc4CL > At4CL3 [32,36]. However, our results revealed a reversed activity profile in *Y. lipolytica*. Among all tested 4CL enzymes, At4CL3 exhibited the highest efficiency for umbelliferone biosynthesis (Figure 4), suggesting its superior compatibility with RgC2′H in the reconstituted biosynthetic pathway. In shake-flask cultivation, the final engineered strain YL-PU accumulated 10.33 ± 0.51 mg/L *p*-coumaric acid, while the umbelliferone titer only reached 3.39 ± 0.15 mg/L (Figure 5), indicating a severe metabolic bottleneck in the downstream conversion of *p*-coumaric acid to umbelliferone. Consistent with this finding, C2′H has been identified as a universal rate-limiting enzyme for heterologous coumarin biosynthesis in microbial cell factories [12]. Future efforts to improve pathway conversion efficiency could be implemented via substrate-binding pocket-directed site-directed mutagenesis of C2′H.

The strain exhibited an extended 23-h lag phase spanning 25–48 h during fed-batch fermentation (Figure 6), presumably attributable to physiological readjustment upon switching the culture media from YPD seed broth to defined mineral medium. A similar phenomenon was also observed in a previous report [33]. The biomass of the strain reached an OD value of 79.13 at 96 h of cultivation, representing an 8-fold increase relative to that obtained from shake-flask culture at 96 h. The titer of umbelliferone in the 84 h fed-batch fermentation broth was 125.52 mg/L, which was 37-fold higher than that measured in the 96 h shake-flask culture. Notably, only trace amounts of *p*-coumaric acid (0.1–0.2 mg/L) were detected in the fed-batch fermentation broth. In contrast, shake-flask culture yielded a significantly higher accumulation of *p*-coumaric acid than umbelliferone (Figure 5). Different culture media were used for the two cultivation modes: a mineral medium was adopted for fed-batch fermentation, while YPD medium was employed for shake-flask culture. A previous study revealed that *Y. lipolytica* could efficiently convert *p*-coumaric acid into unknown metabolites in mineral medium, whereas such metabolic conversion was inefficient in YPD medium [33]. Thus, we speculated that the intermediate *p*-coumaric acid could be largely converted by *Y. lipolytica* during mineral medium-based fed-batch cultivation. It is also plausible that mineral medium, in contrast to rich complex medium, redirects metabolic flux and enhances the conversion of *p*-coumaric acid into umbelliferone. Future studies could focus on identifying the key enzymes responsible for the conversion in *Y. lipolytica*. Knockout of these metabolic enzymes may serve as an effective strategy to block the catabolism of *p*-coumaric acid, thereby substantially improving umbelliferone biosynthesis in *Y. lipolytica* cultured with mineral medium.

## 5. Conclusions

In this work, we engineered the oleaginous yeast *Y. lipolytica* for de novo production of umbelliferone. By screening optimal heterologous enzymes and integrating feedback-insensitive shikimate pathway mutants to boost precursor supply, we constructed strain YL-PU that reached an umbelliferone titer of 125.52 mg/L in 5 L fed-batch fermentation, the highest yeast-based de novo production reported to date, laying a clear framework for further engineering coumarin biosynthesis in this chassis.

## Data Availability

The data presented in this study are available in the main text, figures, tables and Supplementary Materials.

## Supplementary Materials

The following supporting information can be downloaded at https://www.mdpi.com/article/10.3390/biom16101450/s1. Method of Construction of yeast expression vectors, Table S1: The nucleotide sequences of all heterologous genes used in this study, Table S2: Plasmids used and constructed in this study, Table S3: Primers used in this study, Table S4. Strains constructed in this study, Figure S1: Calibration curves of *p*-coumaric acid and umbelliferone standards; Figure S2: UV absorption spectra of *p*-coumaric acid (A) and umbelliferone (B) products in comparison with their corresponding standards; Figure S3: HPLC analysis of culture supernatants from strains expressing FjTAL, ClTAL, and empty-vector control (EV) fed with 10 mM tyrosine; Figure S4: HPLC-UV chromatograms (monitored at 303 nm) comparing the extracellular and intracellular *p*-coumaric acid (p-CA) production in engineered yeast strain YL-P; Figure S5: Comparison of extracellular and intracellular umbelliferone (UMB) titers in engineered yeast strain YL-PU under shake-flask fermentation.

## References

[1] Kornicka A., Balewski L., Lahutta M., Kokoszka J., Chang Y.Y. (2023). Umbelliferone and Its Synthetic Derivatives as Suitable Molecules for the Development of Agents with Biological Activities: A Review of Their Pharmacological and Therapeutic Potential. Pharmaceuticals.

[2] Kim D.J., Jung M.S., Jin H.U., Kim M.S., An C.E. (2024). Anti-Melanogenic Effects of Umbelliferone: In Vitro and Clinical Studies. Molecules.

[3] Yang L., Li S.L., Qin X.Y., Jiang G.F., Chen J.N., Li B.D., Yao X.Y., Liang P.B., Zhang Y., Ding W. (2017). Exposure to Umbelliferone Reduces *Ralstonia solanacearum* Biofilm Formation, Transcription of Type III Secretion System Regulators and Effectors and Virulence on Tobacco. Front. Microbiol..

[4] Wang P.P., Fan Z.J., Wei W.P., Yang C.S., Wang Y., Shen X., Yan X., Zhou Z.H. (2023). Biosynthesis of the Plant Coumarin Osthole by Engineered *Saccharomyces cerevisiae*. ACS Synth. Biol..

[5] Bouillé A., Larbat R., Kumari R., Olry A., Charles C., Nelson D.R., Thornton J., Villard C., Hehn A. (2025). Lineage-specific patterns in the Moraceae family allow identification of convergent P450 enzymes involved in furanocoumarin biosynthesis. New Phytol..

[6] Li X.J., Zhu Z., Han S.L., Zhang Z.L. (2016). Bergapten exerts inhibitory effects on diabetes-related osteoporosis via the regulation of the PI3K/AKT, JNK/MAPK and NF-κB signaling pathways in osteoprotegerin knockout mice. Int. J. Mol. Med..

[7] Kindl H. (1970). The regulation of the L-tyrosine ammonia-lyase activity by phenolic compounds. Hoppe-Seyler’s Z. Fur Physiol. Chem..

[8] Koukol J., Conn E.E. (1961). The metabolism of aromatic compounds in higher plants. IV. Purification and properties of the phenylalanine deaminase of *Hordeum vulgare*. J. Biol. Chem..

[9] Hu Y., Gai Y., Yin L., Wang X., Feng C., Feng L., Li D., Jiang X.N., Wang D.C. (2010). Crystal structures of a *Populus tomentosa* 4-coumarate:CoA ligase shed light on its enzymatic mechanisms. Plant Cell.

[10] Vialart G., Hehn A., Olry A., Ito K., Krieger C., Larbat R., Paris C., Shimizu B., Sugimoto Y., Mizutani M. (2012). A 2-oxoglutarate-dependent dioxygenase from Ruta graveolens L. exhibits p-coumaroyl CoA 2′-hydroxylase activity (C2′H): A missing step in the synthesis of umbelliferone in plants. Plant J. Cell Mol. Biol..

[11] Kim C.Y., Mitchell A.J., Kastner D.W., Albright C.E., Gutierrez M.A., Glinkerman C.M., Kulik H.J., Weng J.K. (2023). Emergence of a proton exchange-based isomerization and lactonization mechanism in the plant coumarin synthase COSY. Nat. Commun..

[12] Rodrigues J.L., Gomes D., Rodrigues L.R. (2022). Challenges in the Heterologous Production of Furanocoumarins in *Escherichia coli*. Molecules.

[13] Balunas M.J., Kinghorn A.D. (2005). Drug discovery from medicinal plants. Life Sci..

[14] Al-Amiery A.A., Musa A.Y., Kadhum A.A.H., Mohamad A. (2011). The Use of Umbelliferone in the Synthesis of New Heterocyclic Compounds. Molecules.

[15] Alipour M., Khoobi M., Moradi A., Nadri H., Moghadam F.H., Emami S., Hasanpour Z., Foroumadi A., Shafiee A. (2014). Synthesis and anti-cholinesterase activity of new 7-hydroxycoumarin derivatives. Eur. J. Med. Chem..

[16] Tyvorskii V.I., Tishchenko I.G., Kukharev A.S. (1986). Synthesis of (+/-)Umbellacton. Zh. Org. Khim..

[17] Yang S.M., Shim G.Y., Kim B.G., Ahn J.H. (2015). Biological synthesis of coumarins in *Escherichia coli*. Microb. Cell Fact..

[18] Lin Y.H., Sun X.X., Yuan Q.P., Yan Y.J. (2013). Combinatorial biosynthesis of plant-specific coumarins in bacteria. Metab. Eng..

[19] Gu Y., Ma J., Zhu Y., Ding X., Xu P. (2020). Engineering *Yarrowia lipolytica* as a Chassis for De Novo Synthesis of Five Aromatic-Derived Natural Products and Chemicals. ACS Synth. Biol..

[20] Xu M., Yang N., Pan J., Hua Q., Li C.X., Xu J.H. (2024). Remodeling the Homologous Recombination Mechanism of *Yarrowia lipolytica* for High-Level Biosynthesis of Squalene. J. Agric. Food Chem..

[21] Schwartz C., Shabbir-Hussain M., Frogue K., Blenner M., Wheeldon I. (2017). Standardized Markerless Gene Integration for Pathway Engineering in *Yarrowia lipolytica*. ACS Synth. Biol..

[22] Gao S.L., Tong Y.Y., Wen Z.Q., Zhu L., Ge M., Chen D.J., Jiang Y., Yang S. (2016). Multiplex gene editing of the *Yarrowia lipolytica* genome using the CRISPR-Cas9 system. J. Ind. Microbiol. Biotechnol..

[23] Virklund A., Jendresen C.B., Nielsen A.T., Woodley J.M. (2023). Characterization of tyrosine ammonia lyases from flavobacterium johnsonian and herpetosiphon aurantiacus. Biotechnol. J..

[24] Brack Y., Sun C.H., Yi D., Bornscheuer U.T. (2022). Discovery of Novel Tyrosine Ammonia Lyases for the Enzymatic Synthesis of *p*-Coumaric Acid. ChemBioChem.

[25] Tork S.D., Nagy E.Z.A., Tomoiaga R.B., Bencze L.C. (2023). Engineered, Scalable Production of Optically Pure L-Phenylalanines Using Phenylalanine Ammonia-Lyase from. J. Org. Chem..

[26] Hou X.M., Shao F.J., Ma Y.M., Lu S.F. (2013). The phenylalanine ammonia-lyase gene family in Salvia miltiorrhiza: Genome-wide characterization, molecular cloning and expression analysis. Mol. Biol. Rep..

[27] Dressen A., Hilberath T., Mackfeld U., Billmeier A., Rudat J., Pohl M. (2017). Phenylalanine ammonia lyase from Arabidopsis thaliana (AtPAL2): A potent MIO-enzyme for the synthesis of non-canonical aromatic alpha-amino acids: Part I: Comparative characterization to the enzymes from Petroselinum crispum (PcPAL1) and Rhodosporidium toruloides (RtPAL). J. Biotechnol..

[28] Gomes D., Rodrigues J.L., Rodrigues L.R. (2024). Step-by-step optimization of a heterologous pathway for de novo naringenin production in *Escherichia coli*. Appl. Microbiol. Biotechnol..

[29] Ren C.Y., Liu Y., Wei W.P., Dai J.B., Ye B.C. (2021). Reconstruction of Secondary Metabolic Pathway to Synthesize Novel Metabolite in *Saccharopolyspora erythraea*. Front. Bioeng. Biotechnol..

[30] Wang H., Liu S.M., Zhang S.B., Fan F.J., Yang C.W., Duan Y.X., Chen Q.M., Liu C. (2025). 4-Coumarate CoA Ligase Family in Soybean Responds to *Heterodera glycines*. Curr. Issues Mol. Biol..

[31] Zhao Y.C., Jian X.Y., Wu J.L., Huang W.C., Huang C.L., Luo J., Kong L.Y. (2019). Elucidation of the biosynthesis pathway and heterologous construction of a sustainable route for producing umbelliferone. J. Biol. Eng..

[32] Bang H.B., Lee Y.H., Kim S.C., Sung C.K., Jeong K.J. (2016). Metabolic engineering of *Escherichia coli* for the production of cinnamaldehyde. Microb. Cell Fact..

[33] Sáez-Sáez J., Wang G., Marella E.R., Sudarsan S., Pastor M.C., Borodina I. (2020). Engineering the oleaginous yeast *Yarrowia lipolytica* for high-level resveratrol production. Metab. Eng..

[34] Barros J., Serrani-Yarce J.C., Chen F., Baxter D., Venables B.J., Dixon R.A. (2016). Role of bifunctional ammonia-lyase in grass cell wall biosynthesis. Nat. Plants.

[35] Larroude M., Nicaud J.M., Rossignol T. (2021). *Yarrowia lipolytica* chassis strains engineered to produce aromatic amino acids via the shikimate pathway. Microb. Biotechnol..

[36] Dunstan M.S., Robinson C.J., Jervis A.J., Yan C.Y., Carbonell P., Hollywood K.A., Currin A., Swainston N., Le Feuvre R., Micklefield J. (2020). Engineering *Escherichia coli* towards de novo production of gatekeeper (2 *S*)-flavanones: Naringenin, pinocembrin, eriodictyol and homoeriodictyol. Syn. Biol..

