# Supplementary material for: Metabolic Engineering of Yarrowia lipolytica for De Novo Biosynthesis of Umbelliferone
Source: Biomolecules. 2026 Oct 4;16(10):1450. doi: 10.3390/biom16101450 (PMC13702494; doi:10.3390/biom16101450)
Supplement: Supplementary file 1 [file biomolecules-16-01450-s001.zip › biomolecules-4545951-supplementary.pdf]

biomolecules

MDPI

Supplementary data for

Metabolic Engineering of *Yarrowia lipolytica* for De Novo Biosynthesis of Umbelliferone

Construction of yeast expression vectors

All engineered plasmids constructed in this study could be classified into three categories, including episomal expression plasmids, CRISPR gene-editing plasmids, and site-specific integrative plasmids.

The empty episomal expression vectors pHRm-URA3 and pYarrow-LEU were modified from the commercial plasmids pHR_AXP_hrGFP and pCASyl , respectively. For the pHRm-URA3 vector, the original endogenous expression cassette of the commercial pHR_AXP_hrGFP backbone was completely replaced with a synthetic dual-gene expression cassette tXPR2-pTEFin-pUAS1B8TEF-tCYC1, while the remaining backbone sequences were fully retained. For the pYarrow-LEU vector, all intrinsic CRISPR-Cas9-associated editing elements (including Cas9 nuclease and gRNA transcription units) on the pCASyl backbone were completely removed and substituted with the identical dual-gene expression cassette tXPR2-pTEFin-pUAS1B8TEF-tCYC1. The modified pYarrow-LEU vec-tor carries the LEU2 auxotrophic selection marker, which serves as an alternative screen-ing marker that is independent of the URA3 marker of the pHRm-URA3.

The recombinant gene expression plasmids constructed in this study included pYarrow-FjTAL, pYarrow-ClTAL, pYarrow-EcaroL, pHRm-AtPAL, pHRm-SmPAL, pHRm-RtPAL-TAL, pHRm-ScARO4-ScARO7, pHRm-YlARO4-YlARO7, pHRm-ScARO4^{K229L}-ScARO7^{G141S}, pHRm-YlARO4^{K221L}-YlARO7^{G139S}, pYarrow-At4CL3, pYarrow-Sc4CL, and pYarrow-Gm4CL4. Except for *YlARO4* and *YlARO7* amplified from *Yarrowia lipolytica*, as well as their homologs *ScARO4* and *ScARO7* amplified from *Saccharomyces cerevisiae*, all other heterologous genes were chemically synthesized with codon optimization tailored to the codon-usage bias of *Y. lipolytica*. The sequences of these synthetic genes are provided in Table S1, and detailed information for all plasmids constructed in this study is summarized in Table S2. All primer sequences used for PCR amplification during plasmid construction are listed in Table S3.

The CRISPR-Cas9 editing plasmids p-CASyl-MFE1 and p-CASyl-D17 were constructed for targeted genomic cleavage. The designed gRNAs of these two plasmids mediate cleavage at the MFE1 locus and the D17 site on chromosome X, respectively.

A series of site-specific integrative plasmids were constructed in this study, including pHRm-MFE1, pHRm-(MFE1)-AtC4H-AtCPR, pHRm-(MFE1)-AtC4H-AtCPR-RtPAL-TAL, pHRm-(MFE1)-AtC4H-AtCPR-RtPAL-TAL-ScARO4^{K229L}-ScARO7^{G141S}-EcaroL, pHRm-D17, pHRm-(D17)-RgC2′H, pHRm-(D17)-AdC2′H, pHRm-(D17)-AtCOSY, pHRm-(D17)-AtCOSY-RgC2′H, and pHRm-(D17)-AtCOSY-RgC2′H-At4CL.

Supplementary Table S1. The nucleotide sequences of all heterologous genes used in this study

**Name**	**Codon optimized sequence**
Ecarol	ATGACTCAACCCCTCTTTTTAATCGGACCTCGTGGATGCGGCAAAACAACAGTAGGCATGGCTTTAGCAGATTCTTTAAACAG<br>GCGCTTCGTAGATACAGACCAATGGCTTCAATCCCAACTCAACATGACCGTCGCCGAAATTGTCGAACGAGAGGAATGGGCAG<br>GTTTCCGTGCACGGGAGACTGCTGCTCTGGAGGCGGTTACTGCCCCTTCGACGGTGATCGCCACAGGAGGAGGCATCATCCTC<br>ACCGAATTCAACAGACACTTTATGCAAAACAATGGTATTGTGGTTTACTTGTGTGCTCCTGTGTCTGTGCTGGTCAACCGACT<br>GCAGGCTGCCCCCGAGGAGGATCTGCGACCCACCCTGACCGGCAAGCCCCTGTCCGAGGAGGTCCAGGAGGTGCTCGAGGAGC<br>GAGACGCCCTTTACAGAGAAGTAGCCCACATTATAATCGACGCCACCAATGAACCCTCCCAAGTAATATCAGAAATCCGTAGT<br>GCCTTAGCCCAGACCATCAATTGCTAA
AtPAL	ATGGAGATTAACGGGGCACATAAGAGTAATGGAGGAGGAGTAGACGCGATGCTATGCGGAGGAGACATAAAAACGAAGAACAT<br>GGTTATTAATGCAGAGGATCCACTGAATTGGGGAGCAGCAGCAGAACAGATGAAGGGGTCCCACTTGGACGAGGTTAAGCGAA<br>TGGTTGCCGAGTTCCGAAAACCGGTGGTGAACCTCGGCGGAGAGACCCTGACCATCGGTCAAGTGGCTGCCATTTCCACCATT<br>GGCAACTCGGTGAAGGTTGAGCTTTCCGAAACTGCACGAGCCGGAGTCAACGCCTCCTCGGATTGGGTCATGGAATCTATGAA<br>CAAGGGCACTGACTCTTACGGAGTAACGACTGGCTTTGGAGCTACCTCTCACCGGCGGACGAAAAATGGAGTTGCTCTCCAGA<br>AGGAGTTGATACGGTTCTTAAACGCTGGCATTTTTGGATCCACCAAGGAAACCTCGCATACCCTTCCTCACTCTGCGACTCGT<br>GCCGCCATGCTGGTGCGAATCAATACTCTTCTGCAAGGTTTCTCTGGCATAAGATTTGAAATACTCGAGGCCATCACGAGCTT<br>CCTAAACAACAACATTACACCTTCTCTACCCCTCCGAGGAACGATCACAGCCTCTGGGGACCTCGTTCCCTTATCCTACATCG<br>CAGGACTCCTCACCGGCCGTCCGAACTCTAAGGCCACTGGACCAAACGGCGAGGCGCTCACAGCGGAGGAGGCTTTCAAGCTT<br>GCTGGGATCTCGTCCGGCTTTTTCGATCTGCAGCCGAAAGAAGGTCTGGCACTTGTCAACGGAACTGCCGTTGGCTCTGGAAT<br>GGCTTCTATGGTTCTGTTTGAGACGAACGTGCTCTCAGTACTAGCTGAAATCTTAAGTGCCGTGTTCGCTGAGGTCATGTCCG<br>GTAAACCCGAGTTCACTGATCATCTGACACACCGACTCAAGCATCACCCTGGTCAAATTGAAGCCGCTGCTATCATGGAACAC<br>ATTCTGGATGGCAGCTCCTACATGAAGCTGGCTCAGAAGCTCCACGAGATGGACCCTCTTCAGAAGCCTAAGCAGGACCGATA<br>TGCTCTTCGGACCTCTCCCCAGTGGTTAGGGCCTCAGATTGAAGTTATCCGATACGCTACTAAGAGTATCGAGAGAGAGATCA<br>ATTCTGTTAACGATAACCCTCTCATTGACGTCTCACGTAACAAAGCCATCCATGGAGGAAACTTTCAAGGAACCCCCATTGGA<br>GTGAGTATGGACAATACACGCTTGGCCATTGCTGCGATCGGCAAGCTGATGTTCGCTCAGTTTTCCGAGCTCGTCAACGACTT<br>TTACAACAATGGCCTGCCCTCGAACTTGACCGCCTCTCGAAACCCCAGCTTGGATTATGGCTTTAAGGGTGCCGAAATTGCCA<br>TGGCATCATACTGTAGCGAGCTGCAGTACCTCGCAAACCCAGTAACTTCGCATGTGCAAAGCGCCGAGCAACACAATCAGGAC<br>GTCAACTCCCTCGGTCTCATCTCAAGCAGAAAAACTTCTGAGGCCGTCGACATCCTGAAGCTGATGTCTACCACGTTCCTAGT<br>CGCCATTTGTCAGGCAGTGGATCTCCGGCACCTTGAGGAGAACCTTCGACAGACCGTGAAGAATACAGTTTCACAGGTGGCCA<br>AGAAGGTGCTGACCACCGGTGTCAACGGTGAGCTGCACCCCTCTCGGTTCTGCGAAAAGGACCTCTTGAAGGTTGTGGACAGG<br>GAACAGGTGTACACCTACGCTGACGACCCATGTTCGGCCACCTATCCCCTCATTCAGAAATTGCGACAGGTAATCGTTGACCA<br>CGCCCTGATCAACGGCGAGTCGGAGAAGAACGCTGTCACATCGATCTTTCATAAGATTGGTGCCTTCGAGGAAGAGCTCAAGG<br>CTGTTCTACCCAAGGAGGTCGAGGCTGCCCGAGCGGCATACGACAACGGTACCTCTGCCATCCCCAATCGCATTAAGGAGTGC<br>AGATCGTATCCTCTGTACCGTTTCGTTAGAGAAGAGCTGGGTACAGAACTTCTCACTGGTGAAAAAGTCACGAGTCCAGGGGA<br>GGAGTTTGATAAAGTATTTACAGCAATATGCGAAGGAAAAATAATAGATCCCATGATGGAATGTTTAAATGAATGGAACGGCG<br>CCCCAATACCTATATGCTAA
SmPAL	ATGACTTTTGTAATTGAACTTGACATGAATGTAACCCTAGACCAACTTGAAGATGCTGCCCGCCAAAGAACCCCTGTAGAACT<br>ATCCGCCCCCGTTAGATCCCGAGTAAGAGCCTCCCGTGATGTATTGGTCAAATTTGTGCAGGATGAGCGAGTGATATACGGTG<br>TCAATACGTCTATGGGTGGATTCGTCGATCACCTGGTCCCCGTTTCCCAGGCCCGACAGCTTCAGGAGAATTTGATTAACGCC<br>GTCGCCACAAACGTTGGTGCGTACCTTGACGACACGACTGCCCGAACCATCATGCTCAGCAGAATCGTCTCCTTGGCCAGAGG<br>AAACTCGGCCATTACGCCAGCTAACCTGGACAAGCTTGTAGCTGTTCTCAATGCCGGCATTGTTCCTTGTATCCCTGAGAAAG<br>GGTCTCTTGGTACGTCCGGTGACTTGGGTCCCCTTGCTGCCATCGCTCTGGTTTGTGCGGGCCAGTGGAAGGCGAGATACAAT

	GGCCAGATCATGCCTGGGCGACAAGCCCTGTCTGAAGCAGGAGTGGAACCCATGGAGCTTAGTTATAAGGATGGCCTTGCCCTGATTAATGGTACCTCTGGCATGGTCGGCTTGGGCACTATGGTCCTGCAGGCTGCACGACGTCTGGTGGATCGGTACCTCCAGGTGTCTGCTCTCTCCGTCGAGGGTCTCGCTGGAATGACCAAGCCCTTTGATCCCCGGGTCCATGGCGTAAAGCCCCACCGAGGACAGCGCCAAGTGGCAAGCCGGCTGTGGGAAGGTCTGGCCGACTCCCACTTGGCTGTGAACGAGCTGGATACTGAGCAGACCCTGGCCGGAGAGATGGGAACCGTCGCAAAGGCTGGTAGTCTGGCTATTGAAGATGCCTACTCGATCAGATGCACACCCCAGATCCTTGGTCCCGTGGTGGACGTGCTTGATCGAATTGGAGCAACATTGCAGGACGAGCTCAACTCTTCTAACGACAACCCTATAGTGCTGCCTGAAGAGGCAGAGGTTTTCCACAACGGACACTTCCATGGACAATACGTGGCCATGGCCATGGACCATCTCAACATGGCGCTGGCAACTGTGACGAATCTCGCCAACCGACGAGTCGACCGATTCCTCGACAAGAGCAACTCCAACGGCCTGCCGGCGTTCCTCTGCCGTGAGGACCCGGGTCTTCGACTGGGCCTCATGGGAGGCCAATTCATGACTGCTTCCATCACCGCTGAGACCCGAACTCTGACTATCCCTATGTCGGTACAGTCTCTGACCTCTACAGCTGACTTTCAGGACATTGTCTCCTTTGGTTTCGTTGCCGCTCGACGGGCGAGGGAGGTGCTCACCAACGCTGCTTACGTAGTTGCTTTTGAACTGCTCTGTGCCTGCCAGGCCGTTGACATACGAGGCGCCGATAAGTTGTCAAGCTTCACACGCCCCCTATATGAGAGAACCCGTAAGATTGTCCCCTTTTTTGACCGAGACGAGACCATCACAGATTACGTAGAGAAACTAGCAGCAGACTTAATAGCAGGAGAACCAGTAGACGCAGCCGTAGCAGCCCATTAG
RtPAL-TAL	ATGGCCCCCAAGTCTTGACTCAATCTCTCACTCCTTCGCAAATGGTGTCGCTTCTGCTAAACAGGCTGTTAACGGTGCTTCAACAAACCTCGCTGTCGCTGGATCTCATTTACCCACTACTCAAGTTACGCAGGTGGATATTGTTGAGAAAATGCTAGCTGCCCCCACGGATTCGACGCTAGAGCTCGACGGTTACAGCCTCAACCTTGGCGACGTGGTGTCCGCGGCCAGGAAGGGCCGTCCTGTGCGAGTGAAGGATTCAGACGAGATTCGATCTAAGATAGACAAGAGCGTTGAATTCTTGAGGAGCCAGCTGTCCATGTCAGTTTACGGAGTAACGACTGGCTTTGGAGGATCAGCGGACACCCGGACAGAAGATGCCATCTCTCTGCAGAAGGCGCTGCTTGAACACCAATTGTGCGGTGTCTTACCCTCCTCTTTCGACAGCTTTCGACTCGGACGAGGTCTTGAGAACTCTCTACCCCTGGAAGTTGTTCGAGGCGCGATGACCATTCGAGTCAATTCGCTCACCCGCGGACACAGTGCTGTGCGCCTGGTCGTCCTGGAGGCCCTGACTAACTTCCTTAACCACGGCATCACCCCAATCGTTCCCCTCCGAGGAACGATCTCCGCCTCTGGGGACCTCTCCCCCTTATCCTACATCGCAGCAGCCATCAGTGGCCACCCGGACTCGAAGGTGCATGTAGTGCACGAGGGAAAGGAGAAGATCCTTTACGCTCGCGAGGCCATGGCTCTCTTCAACTTGGAGCCCGTGGTGCTGGGACCGAAAGAAGGTCTGGGGCTTGTCAACGGAACTGCCGTTTCGGCCTCAATGGCCACACTGGCCTTACACGACGCTCACATGCTCAGCCTTCTGTCCCAGTCTCTCACTGCCATGACTGTGGAGGCTATGGTAGGCCATGCCGGATCCTTCCACCCTTTTCTCCATGATGTCACTAGACCTCACCCTACACAAATCGAGGTTGCCGGCAATATTCGGAAGTTACTCGAGGGCTCTCGGTTCGCAGTCCACCATGAGGAAGAGGTCAAAGTCAAGGACGACGAGGGTATCCTGCGACAGGACCGATATCCTCTTCGGACCTCTCCCCAGTGGTTAGGGCCTCTGGTGAGTGATCTCATCCACGCTCATGCCGTGCTTACCATCGAAGCAGGCCAGTCTACAACCGATAACCCTCTCATTGACGTCGAAAACAAAACTTCCCATCATGGAGGAAACTTTCAAGCCGCTGCTGTTGCGAACACTATGGAGAAGACTCGACTGGGCTTGGCACAGATCGGCAAGCTGAACTTCACCCAGCTAACCGAGATGCTCAATGCTGGAATGAATAGAGGCCTGCCCTCGTGTCTTGCTGCTGAAGACCCCTCTCTTTCTTACCATTGCAAAGGTCTGGACATCGCTGCCGCTGCCTACACTTCAGAGTTGGGTCACCTCGCAAACCCAGTAACTACCCATGTTCAACCTGCCGAAATGGCAAACCAGGCCGTCAACTCCCTCGCTCTGATTAGCGCCCGAAGAACAACCGAGTCTAATGATGTGTTGTCTCTGTTGCTAGCCACCCATCTGTACTGTGTGCTACAGGCGATAGATCTGAGGGCCATTGAGTTCGAGTTTAAGAAGCAGTTCGGGCCCGCCATTGTTTCACTTATTGACCAACACTTTGGCTCCGCCATGACCGGATCGAATCTACGAGACGAGCTCGTCGAGAAGGTGAACAAGACCCTGGCTAAACGTCTGGAGCAGACGAACTCGTACGACCTGGTTCCTCGGTGGCACGACGCTTTTTCCTTCGCTGCTGGCACAGTTGTGGAGGTGCTGTCTTCAACGTCACTCTCGCTGGCCGCGGTCAACGCATGGAAGGTCGCTGCCGCCGAGTCGGCTATTTCCCTGACCCGACAGGTCAGAGAGACGTTCTGGTCTGCCGCATCTACTTCTTCCCCAGCCCTTTCGTATCTATCGCCCCGAACACAGATTTTGTACGCTTTCGTTAGAGAAGAGCTGGGTGTCAAGGCACGACGCGGCGATGTTTTTCTGGGTAAACAGGAAGTCACAATAGGATCAAACGTCAGTAAAATTTACGAAGCAATAAAGTCAGGAAGAATCAACAACGTACTATTAAAAATGCTAGCATAA
FjTAL	ATGAACACCATCAACGAGTACCTGTCCCTAGAAGAGTTTGAAGCAATTATATTTGGTAACCAGAAAGTAACAATTAGTGATGTTGTAGTAAACCGTGTCAATGAATCATTCAACTTCCTTAAAGAATTCTCTGGAAATAAGGTGATATACGGTGTCAATACGGGTTTCGGGCCTATGGCCCAATATAGAATTAAAGAATCTGATCAAATTCAACTCCAGTACAACTTGATCCGATCCCACTCTTCTGGCACCGGCAAGCCTCTTTCGCCCGTGTGCGCTAAGGCAGCCATCCTCGCCCGACTCAACACCCTGTCTCTGGGGAACTCCGGCGTCCACCCGTCCGTGATCAACCTGATGTCGGAGCTCATCAACAAGGACATCACTCCCCTGATCTTTGAGCACGGTGGCGTTGGGG

	CTTCCGGTGACTTGGTTCAGCTGTCGCATCTGGCTCTGGTGTTGATAGGAGAAGGCGAGGTCTTCTACAAGGGCGAACGAAGA<br>CCTACCCCGGAGGTTTTTGAAATCGAGGGTCTCAAGCCAATTCAGGTGGAGATTCGGGAGGGCCTTGCCCTGATTAATGGTAC<br>CTCTGTCATGACTGGGATCGGAGTTGTAAACGTCTATCACGCAAAGAAGCTCCTGGATTGGTCTCTCAAATCTAGCTGTGCTA<br>TCAACGAACTCGTCCAGGCATATGACGACCACTTTTCTGCCGAGCTTAACCAGACCAAGCGTCATAAAGGACAGCAGGAGATC<br>GCTCTCAAGATGCGCCAGAACCTCTCAGACTCGACACTTATCCGAAAACGAGAGGATCACTTATACTCGGGAGAGAACACGGA<br>AGAGATTTTCAAGGAGAAGGTCCAGGAGTACTACTCGCTGCGTTGTGTTCCCCAGATCCTTGGTCCCGTGCTCGAGACCATCA<br>ACAACGTAGCGTCAATCCTAGAGGACGAATTCAACTCAGCAAACGACAACCCTATAATAGACGTGAAGAATCAGCATGTTTAC<br>CATGGCGGAAATTTTCATGGAGATTACATTAGTCTGGAGATGGACAAGCTGAAGATCGTCATCACCAAACTGACAATGTTGGC<br>TGAGCGACAGCTCAATTACCTGCTCAACTCGAAGATCAATGAGCTGCTGCCTCCCTTTGTAAACCTGGGAACCCTTGGATTCA<br>ACTTTGGAATGCAAGGAGTGCAGTTCACAGCCACGTCAACTACCGCCGAATCCCAGATGCTGTCCAACCCCATGTACGTGCAC<br>TCAATTCCAAACAACAATGATAACCAGGACATTGTCTCCATGGGTACTAACTCTGCTGTGATTACTAGCAAGGTTATTGAGAA<br>TGCCTTCGAGGTGCTGGCCATTGAAATGATCACAATTGTGCAGGCGATTGATTACCTGGGCCAGAAGGACAAGATTTCCTCCG<br>TCTCCAAGAAGTGGTACGACGAAATCCGAAATATTATCCCCACGTTTAAAGAAGACCAAGTTATGTATCCATTTGTTCAAAAA<br>GTTAAAGATCACCTTATCAACAATTAG
ClTAL	ATGAAGATCAACAACTTTTTAGGCCTCAAAGACTTCCAGAAAATCATCATCGAAAACGGCAAAATTGAATTAGACGAATCACT<br>ATTAGACCGAGTAGAAGCATCATTCCAATTCCTTAAAGAATTCTCTCGTAATAAGGTGATATACGGTGTCAATACGGGTTTCG<br>GGCCTATGGCCCAATTTAAAATTTCAGATGAAGACACCCACCAACTCCAGTACAACTTGATCCGATCCCACTCTTCTGGCATC<br>GGAAACCCCCTTCCCGCACAAGAGGTCAAGGCCTGCATGCTCGCCCGACTCAACACCCTGTCTCTGGGGAACTCCGGCGTCCA<br>CCAATCCGTGGTCCACCTTTTGAAGGAGCTCATCAACCGCGACATCACTCCCCTGATCTTTGAGCACGGTGGCGTTGGGGCTT<br>CCGGTGACTTGGTTCAGCTGGCGCATCTGGCTCTGGTGTTGATAGGAGAAGGCGAGGTCTTCTACAAGGGCGAACGAACCTCC<br>ACCAAGGAGGTTTTTGAAATCGAGGGTCTCGAGCCAATTCAGGTGGAGATTCGGGAGGGCCTTGCCCTGATGAATGGTACCTC<br>TGTCATGTCTGGGATCGGAGTTGTAAACGCCTACAAGGCAAATCAGCTGACCGACATCAGCATAAAGCTGAGCTGTGCTATCA<br>ACGAAATTGTCCAGGCATATGACGACCACCTGTCTGAAGCTCTCAACGGCACCAAAAAGCACTATGGCCAACAGAAGGTGGCT<br>GAGCGTATGCGAGCCCATCTCGCCGACAGCAAGCTTATCCGAAAACGAGAGGATCACTTATACACTCACTTCGAGGAGCAGGA<br>AAAGGTCTTCAAGGAGAAGGTCCAGGAGTACTACTCGCTGCGTTGTGTTCCCCAGATCCTTGGTCCCGTGCTCGATACGCTCG<br>AGTATACTGAGACAGTCCTGGAAAACGAAATCAACTCAGCAAACGACAACCCTATAATAAACGTCGAGGACAAGCATGTTTAC<br>CATGGCGGAAATTTTCATGGAGATTACATTAGTCTGGAGATGGACAAGCTGAAGATCGTCGTTACCAAACTGACAATGTTGGC<br>TGAGCGACAGCTCAATTACCTGCTCAACGCTAAGATCAATGAGATTCTGCCTCCCTTTGTAAACCTGGGAAAACTTGGATTCA<br>ACTTTGGAATGCAAGGAGTGCAGTTCACAGCCACGTCAACTACCGCCGAATCCCAGATGCTGTCCAACTCGATGTACGTGCAC<br>TCAATTCCAAACAACAATGATAACCAGGACATTGTCTCCATGGGTACTAACGCTGCCGTGATCTGTCGAAAGGTTATTGAGAA<br>TGCCTTCGAGGTGCTGGCCATTGAAGCCATTACTATTATCCAGGCAGTGGAGTACCTTGGTTTCCAGGATAAGGTGTCCTCGT<br>CCACAAAGGAGCTTTACGACGAAATCCGAAAAATAATCCCCGCATTTAGTAACGACATGGTTATGTATCCATATTTAGAAGAA<br>GTTAAAAAATACCTTAAAGCAATGTAA
AtC4H	ATGGACTTATTATTACTTGAAAAATCATTAATCGCCGTATTTGTCGCCGTCATTCTAGCAACCGTCATATCAAAACTAAGAGG<br>AAAAAAATTGAAACTACCCCCCGGACCTATCCCTATACCTATTTTCGGTAACTGGTTGCAGGTGGGGGACGATCTGAACCACC<br>GAAACCTTGTCGACTACGCGAAGAAGTTCGGTGATCTCTTCCTGCTCAGGATGGGACAGCGCAACTTGGTTGTTGTATCATCT<br>CCGGACCTTACCAAAGAAGTTCTGCTGACTCAAGGCGTGGAGTTTGGATCCCGCACAAGAAACGTGGTGTTCGATATCTTCAC<br>TGGCAAGGGTCAGGACATGGTCTTCACCGTCTACGGAGAGCATTGGAGAAAAATGCGTAGAATCATGACCGTGCCCTTTTTCA<br>CCAACAAGGTGGTCCAGCAGAATAGAGAAGGCTGGGAGTTTGAGGCCGCCAGTGTGGTTGAGGATGTCAAGAAGAACCCCGAC<br>TCCGCCACTAAGGGTATTGTGCTGAGAAAGCGTCTGCAGCTTATGATGTACAACAACATGTTTCGAATCATGTTCGATCGACG<br>TTTCGAGTCTGAAGACGACCCACTATTTCTTCGGCTGAAGGCTCTCAACGGAGAACGGTCCAGGTTAGCTCAGTCTTTTGAGT<br>ACAACTATGGCGATTTTATTCCCATTCTGCGGCCCTTCCTGCGAGGATACCTCAAGATTTGCCAAGATGTGAAGGACCGTCGA<br>ATTGCTCTGTTCAAGAAATACTTTGTGGATGAACGAAAGCAGATTGCCAGTTCCAAGCCCACAGGATCGGAAGGCCTCAAGTG<br>CGCCATTGACCACATTCTCGAGGCTGAACAGAAGGGCGAGATCAACGAGGACAACGTCTTGTACATCGTAGAGAACATTAATG

	TGGCAGCCATCGAAACGACGCTCTGGAGCATTGAGTGGGGAATCGCTGAGCTGGTGAATCATCCTGAGATTCAGAGCAAGCTC<br>CGAAACGAGCTTGACACCGTTCTCGGTCCAGGAGTGCAGGTCACAGAGCCTGACCTGCATAAGCTGCCCTACTTGCAGGCGGT<br>TGTCAAGGAAACTCTGCGACTCCGGATGGCTATTCCTTTGCTGGTCCCCCACATGAATCTCCACGACGCCAAACTGGCCGGTT<br>ATGATATCCCCGCCGAGTCCAAAATCTTAGTTAACGCATGGTGGCTCGCCAATAACCCCAACTCTTGGAAGAAGCCGGAGGAG<br>TTCCGGCCCGAGCGATTCTTTGAAGAGGAGTCGCACGTCGAGGCAAACGGTAACGACTTCCGATATGTGCCTTTCGGCGTTGG<br>ACGACGATCGTGTCCAGGCATCATCCTGGCTCTTCCTATCTTGGGTATCACCATTGGCCGAATGGTGCAAAATTTCGAGCTCC<br>TGCCTCCTCCAGGACAATCAAAAGTTGACACATCGGAAAAAGGGGGCCAATTCAGTTTACACATTCTTAATCACTCTATAATA<br>GTTATGAAACCACGCAATTGCTAA
AtCPR	ATGACCTCAGCACTTTACGCCTCAGACCTCTTCAAGCAATTAAAATCAATAATGGGAACCGATAGCCTTTCAGACGACGTAGT<br>ACTTGTAATCGCCACAACCTCCTTAGCCCTAGTCGCCGGATTTGTTGTGCTGCTGTGGAAGAAAACTACCGCCGACCGGTCTG<br>GCGAGCTTAAGCCACTGATGATTCCTAAGTCTCTGATGGCGAAGGATGAAGACGACGATCTAGATCTCGGATCGGGCAAGACC<br>CGTGTTTCTATTTTCTTTGGAACGCAGACCGGCACGGCCGAGGGATTCGCCAAGGCCCTTTCCGAGGAGATTAAGGCCAGATA<br>CGAGAAGGCTGCTGTTAAGGTTATCGATCTGGATGATTACGCTGCAGACGATGACCAATATGAGGAAAAGCTGAAGAAGGAGA<br>CACTGGCCTTCTTCTGTGTTGCCACGTACGGTGACGGAGAGCCCACAGACAACGCTGCTCGATTCTACAAGTGGTTTACCGAA<br>GAGAACGAGCGAGACATTAAACTGCAGCAGTTGGCGTACGGCGTCTTTGCTCTGGGCAACAGACAATACGAGCACTTCAACAA<br>GATTGGGATTGTTTTGGACGAGGAGCTGTGTAAAAAGGGTGCCAAGCGGTTGATCGAGGTGGGCCTGGGAGATGACGACCAGT<br>CTATTGAAGATGATTTCAACGCCTGGAAGGAGTCGCTGTGGTCAGAGCTGGACAAGCTGCTCAAGGACGAGGACGACAAGAGC<br>GTTGCTACCCCCTATACCGCGGTCATTCCAGAATACAGAGTGGTGACCCATGACCCTCGTTTCACCACTCAGAAATCGATGGA<br>GTCCAACGTGGCCAACGGCAATACAACCATCGACATCCACCACCCCTGTCGGGTGGATGTGGCTGTCCAGAAGGAGTTGCACA<br>CCCATGAGTCTGATAGGAGTTGTATTCACTTAGAATTTGACATCTCCCGAACAGGCATCACATACGAAACTGGAGACCACGTT<br>GGCGTGTACGCCGAAAACCACGTGGAGATTGTCGAGGAGGCCGGAAAGCTTCTGGGTCACTCGCTCGACCTCGTGTTCTCAAT<br>CCATGCGGATAAGGAGGACGGTTCTCCTCTCGAGAGTGCTGTGCCCCCTCCCTTCCCCGGCCCTTGTACTCTTGGAACCGGAC<br>TCGCTCGGTATGCTGACCTGCTGAACCCTCCTCGAAAGTCCGCCTTAGTCGCCTTGGCTGCCTACGCCACTGAACCCTCGGAG<br>GCTGAAAAGCTCAAGCATCTGACTAGCCCCGATGGAAAAGACGAATACTCACAGTGGATTGTGGCGTCCCAGCGGTCACTTCT<br>TGAAGTCATGGCAGCATTTCCCTCTGCCAAGCCCCCACTCGGTGTTTTCTTCGCCGCCATAGCTCCCCGATTGCAGCCGCGAT<br>ATTACTCGATTTCAAGCTCTCCCCGCCTGGCTCCTTCCCGAGTACATGTTACCTCTGCTCTTGTATACGGACCAACCCCAACT<br>GGTAGGATCCACAAAGGTGTCTGCTCGACGTGGATGAAAAACGCAGTCCCTGCTGAGAAGTCGCATGAATGCAGCGGGGCTCC<br>AATCTTTATCCGGGCAAGCAATTTTAAACTTCCCTCCAACCCTTCTACACCGATCGTCATGGTTGGGCCGGGT<br>ACCGGTTTGGCCCCCTTTCGAGGATTTCTGCAGGAGCGAATGGCTCTCAAGGAAGACGGCGAAGAGCTCGGCTCTTCCCTGCT<br>ATTCTTCGGTTGCCGCAATAGACAAATGGATTTCATCTACGAGGATGAGCTCAATAACTTTGTCGACCAGGGAGTGATTTCTG<br>AGCTGATTATGGCCTTCTCGCGAGAAGGCGCCCAGAAAGAGTACGTGCAGCACAAGATGATGGAGAAGGCAGCTCAAGTCTGG<br>GATCTTATCAAGGAGGAGGGTTATCTCTACGTTTGCGGCGACGCCAAAGGTATGGCCCGAGATGTGCATCGTACCCTACACAC<br>GATAGTACAGGAACAGGAGGGAGTAAGCAGTAGTGAAGCAGAGGCAATAGTTAAGAAATTACAAACAGAGGGGAGATATTTAA<br>GGGACGTATGGTAA
AtCOSY	ATGGCCACACTTGAAATCACCGACATCGCATTAGTACAACCCTCCCACCAACCTCTCTCAAACGACCAGACCCTTTCTCTCTC<br>CCATCTTGATAATGACAACAACCTCCACGTTTCTTTCCGATACCTCAGAGTCTACTCTTCCAGCTCGTCCACGGTGGCGGGGG<br>AGAGTCCGTCTGCTGTGGTTTCCGCCTCTCTGGCTACTGCACTGGTCCACTACTATCCTCTGGCCGGCTCGCTGCGACGCTCG<br>GCTTCTGATAACAGATTTGAGCTTCTGTGCTCAGCCGGACAGAGTGTTCCCCTGGTGAACGCCACCGTCAACTGTACGCTGGA<br>GTCCGTCGGCTACCTGGACGGTCCCGACCCTGGTTTTGTTGAGCGACTAGTGCCCGACCCCACACGAGAGGAGGGCATGGTCA<br>ACCCCTGTATCTTGCAGGTCACCATGTTCCAGTGTGGGGGCTGGGTACTCGGAGCATCTATTCACCACGCCATCTGTGACGGC<br>TTAGGAGCCTCCCTTTTCTTCAACGCTATGGCTGAGCTGGCGCGCGGAGCTACCAAAATCTCCATTGAACCGGTGTGGGACCG<br>TGAGCGACTGCTGGGACCCCGGGAGAAGCCTTGGGTTGGCGCCCCCGTGAGAGACTTCCTCTCACTCGACAAGGACTTTGATC<br>CCTACGGTCAAGCCATTGGGGATGTGAAGCGAGATTGCTTCTTTGTCACTGACGATTCACTGGATCAGCTTAAAGCGCAGCTC<br>CTCGAAAAGAGCGGCTTGAACTTTACCACTTTCGAGGCCCTCGGTGCTTACATCTGGCGGGCCAAGGTGCGAGCTGCCAAGAC

	TGAAGAGAAGGAAAACGTTAAGTTTGTGTACTCCATCAACATTCGTCGACTCATGAATCCTCCTCTTCCCAAGGGTTATTGGG<br>GAAACGGATGCGTGCCTATGTATGCGCAAATCAAGGCTGGTGAGCTCATCGAGCAGCCGATCTGGAAGACAGCCGAACTCATT<br>AAGCAGTCGAAGTCTAACACGTCTGACGAATACGTCAGATCGTTCATTGATTTCCAGGAGCTGCACCATAAGGATGGAATAAA<br>TGCCGGTACCGGTGTGACCGGCTTCACAGACTGGCGGTACCTGGGCCATTCCACCATTGACTTTGGCTGGGGAGGACCTGTGA<br>CTGTTCTTCCATTGAGCAATAAACTG<br>CTTGGATCTATGGAGCCATGCTTCTTCCTTCCTTACTCGACCGACGCAGCTGCTGGCTCTAAAAAGGACTCAGGTTTTAAAGT<br>ACTAGTTAATTTAAGAGAAAGCGCTATGCCCGAGTTCAAAGAAGCAATGGACAAATTTCACAAAGGAGAGTTCGCATTATCAT<br>AA
At4CL3	ATGATCACCGCCGCACTCCACGAACCCCAGATCCACAAGCCGACAGACACCTCCGTAGTATCAGATGACGTCCTCCCACACTC<br>TCCACCCACTCCCAGAATTTTCCGATCAAAGCTCCCAGACATTGACATACCGAACCACCTACCCCTCCATACGTACTGCTTTG<br>AGAAGCTCTCTTCTGTTAGTGACAAGCCATGCCTCATTGTAGGGAGTACCGGCAAGTCGTACACTTACGGCGAGACACACCTC<br>ATCTGCCGTCGAGTGGCCTCGGGTCTTTACAAGTTGGGAATCCGAAAGGGTGACGTCATTATGATTCTGTTGCAAAACAGTGC<br>CGAGTTCGTCTTCTCTTTCATGGGAGCTAGTATGATTGGTGCTGTTTCAACCACAGCCAACCCGTTCTACACGTCTCAAGAGC<br>TTTACAAGCAGCTCAAGAGCTCTGGTGCCAAGCTCATCATTACCCACTCACAGTACGTCGACAAACTAAAAAACCTTGGAGAA<br>AATCTGACCCTCATCACCACTGACGAGCCCACCCCGGAGAATTGTCTGCCGTTTTCCACTCTAATTACCGATGATGAGACAAA<br>TCCTTTCCAGGAAACCGTCGATATTGGCGGCGACGATGCCGCAGCTCTGCCCTTCTCTAGTGGCACAACTGGACTTCCCAAGG<br>GAGTCGTTCTCACGCACAAGTCTCTGATCACCTCTGTTGCCCAACAGGTTGATGGTGACAACCCAAACTTGTACCTCAAGTCT<br>AATGACGTCATCCTATGCGTCCTGCCCCTGTTCCATATCTACTCGCTGAATAGCGTGCTGCTCAACTCGCTCCGCTCCGGTGC<br>TACCGTTCTGCTGATGCACAAGTTTGAGATTGGCGCCCTGCTAGATCTGATTCAGCGCCATAGGGTTACGATCGCTGCTCTCG<br>TTCCTCCTCTGGTGATCGCGCTCGCCAAGAACCCGACTGTCAATTCCTATGATCTGTCCTCTGTGCGATTTGTCCTCAGTGGC<br>GCAGCGCCCCTCGGAAAGGAGCTCCAGGATTCCTTGAGGAGAAGACTTCCCCAGGCAATTCTCGGCCAGGGTTACGGCATGAC<br>GGAGGCTGGTCCTGTGCTTTCGATGTCACTGGGTTTCGCCAAGGAGCCCATACCTACCAAGTCTGGTTCGTGTGGAACCGTTG<br>TCAGAAACGCCGAACTCAAGGTGGTCCATCTCGAGACCCGACTGTCGCTTGGCTACAACCAGCCTGGAGAGATTTGTATCCGA<br>GGCCAACAGATTATGAAAGAGTACCTTAACGACCCTGAGGCCACGTCGGCGACTATCGACGAGGAGGGTTGGTTGCACACTGG<br>CGATATAGGTTACGTGGACGAGGACGACGAAATCTTCATCGTCGACCGACTCAAGGAGGTCATTAAGTTCAAGGGCTTTCAAG<br>TGCCCCCTGCTGAACTGGAGTCGCTATTGATTAACCACCATTCGATTGCCGACGCTGCCGTCGTCCCTCAGAACGATGAAGTG<br>GCTGGAGAAGTCCCCGTGGCCTTCGTCGTAAGGTCCAACGGCAACGACATCACCGAAGAGGACGTTAAGGAGTATGTTGCCAA<br>ACAGGTCGTCTTCTATAAGCGATTGCATAAGGTTTTCTTCGTCGCATCCATTCCTAAATCCCCATCAGGAAAA<br>ATATTAAGAAAAGATCTCAAAGCCAAATTATGTTAG
Sc4CL	ATGTTTAGAAGCGAATATGCCGATGTCCCCCCTGTTGACTTACCAATCCACGATGCTGTACTAGGAGGAGCCGCCGCCTTTGG<br>ATCCACGCCCGCGCTGATTGATGGAACTGATGGAACTACACTAACCTACGAGCAGGTTGACAGATTTCACCGACGCGTTGCCG<br>CCGCTCTCGCCGAGACCGGCGTGCGAAAGGGTGACGTCTTGGCCCTGCATAGCCCCAACACAGTGGCTTTCCCATTGGCGTTC<br>TACGCCGCCACACGAGCTGGCGCAAGCGTTACCACGGTTCATCCCCTTGCCACTGCTGAGGAATTCGCCAAGCAGTTGAAAGA<br>TTCCGCTGCTCGGTGGATTGTCACCGTCTCTCCTCTTTTAAGTACCGCGCGGCGTGCTGCTGAGCTGGCTGGTGGAGTGCAGG<br>AGATCCTGGTCTGTGACTCAGCCCCAGGACACAGATCCCTGGTGGACATGCTGGCCTCTACTGCCCCCGAGCCCTCCGTGGCC<br>ATAGACCCCGCCGAAGATGTAGCAGCTCTGCCTTATTCTAGTGGCACAACTGGAACCCCCAAGGGAGTCATGCTCACCCATCG<br>ACAGATCGCTACCAATCTGGCACAGCTTGAGCCATCAATGCCATCGGCTCCCGGGGATCGGGTGCTTGCCGTTCTGCCCTTCT<br>TTCACATCTACGGCCTGACCGCTCTTATGAACGCTCCTCTGCGTCTGGGCGCTACTGTGGTTGTTCTTCCCAGATTTGATCTC<br>GAGCAATTCCTCGCTGCCATTCAGAACCACCGAATCACATCTTTATACGTCGCTCCTCCCATCGTGCTCGCGCTCGCCAAGCA<br>CCCTCTCGTCGCAGACTATGATCTGTCCTCTCTGCGATACATTGTGTCTGCCGCAGCGCCCCTCGATGCTCGACTGGCGGCTG<br>CCTGCAGTCAGCGACTTGGCTTGCCTCCTGTTGGCCAGGCATACGGCATGACGGAGTTAAGCCCTGGAACACACGTGGTGCCG<br>CTAGACGCCATGGCTGACGCACCCCCTGGTACCGTGGGCCGGCTCATCGCTGGAACTGAAATGCGTATTGTTTCGCTCACTGA<br>TCCCGGCACCGACCTCCCTGCAGGTGAATCGGGAGAAATCCTGATTCGCGGCCCCCAGATTATGAAAGGATACCTTGGAAGAC

	CGGATGCCACCGCCGCCATGATCGACGAGGAGGGTTGGTTGCACACTGGCGATGTGGGTCACGTAGACGCCGACGGGTGGCTG<br>TTCGTGGTCGACCGAGTGAAGGAGCTCATTAAGTACAAGGGCTTTCAAGTGGCCCCTGCTGAACTGGAGGCACATCTCTTGAC<br>CCACCCCGGAGTTGCCGACGCTGCCGTCGTCGGTGCATATGACGATGACGGGAACGAGGTCCCGCATGCCTTCGTCGTAAGGC<br>AACCTGCCGCTCCCGGTCTGGCCGAGTCCGAGATCATGATGTACGTAGCCGAGCGAGTCGCCCCATACAAGAGAGTTAGAAGA<br>GTTACATTCGTAGACGCAGTCCCCAGAGCAGCTTCAGGAAAAATATTAAGACGCCAATTAAGAGAACCAAGATAG
Gm4CL4	ATGATCACCCTCGCCCCCTCTCTCGACACCCCCAAAACAGACCAGAACCAAGTTTCCGACCCCCAGACCTCTCACGTATTCAA<br>ATCAAAGCTCCCAGACATTCCCATCTCCAACCACCTACCCCTCCATAGTTACTGTTTCCAAAACCTCTCCCAGTTTGCACACC<br>GACCATGCCTCATTGTAGGGCCAGCGTCCAAAACATTCACCTACGCCGACACACACCTCATCTCTTCTAAGATCGCTGCCGGT<br>TTGTCCAACCTCGGTATCTTAAAGGGTGACGTCGTGATGATTCTGTTGCAAAACAGTGCCGATTTCGTCTTCTCTTTCTTAGC<br>CATTAGTATGATTGGTGCTGTTGCAACCACAGCCAACCCGTTCTACACGGCGCCTGAGATCTTCAAACAGTTTACTGTGAGCA<br>AAGCCAAGCTCATCATTACCCAGGCGATGTACGTCGACAAACTACGAAACCACGACGGAGCTAAGCTGGGCGAGGACTTTAAG<br>GTTGTTACTGTTGACGACCCTCCGGAGAATTGTCTGCACTTTTCGGTCCTTTCAGAGGCTAATGAGTCTGACGTTCCCGAGGT<br>CGAGATCCACCCCGATGATGCAGTAGCTATGCCCTTCTCTAGTGGCACAACTGGACTCCCAAGGGAGTCATTCTCACGCACAA<br>GTCTCTGACCACCTCTGTTGCCCAACAGGTTGATGGTGAGAACCCAAACTTGTACCTCACCACTGAGGACGTCTTGCTATGCG<br>TCCTGCCCCTGTTCCATATCTTCTCGCTGAATAGCGTGCTGCTCTGCGCCCTGAGAGCCGGCTCTGCCGTTCTGCTGATGCAG<br>AAGTTTGAGATTGGCACCCTACTTGAGCTGATTCAGCGCCATAGGGTTTCTGTGGCTATGGTCGTTCCTCCTCTGGTGCTCGC<br>GCTCGCCAAGAACCCGATGGTTGCTGACTTCGATCTGTCCTCTATCCGGCTGGTCCTCAGTGGCGCAGCGCCCCTCGGAAAGG<br>AGCTCGAGGAAGCTCTCCGAAACCGAATGCCTCAGGCTGTGCTCGGCCAGGGTTACGGCATGACGGAGGCTGGTCCTGTGCTT<br>TCGATGTGCCTGGGTTTCGCCAAGCAGCCCTTTCAAACCAAGTCTGGTTCGTGTGGAACCGTTGTCAGAAACGCCGAACTCAA<br>GGTGGTCGACCCCGAAACTGGTCGTTCGCTTGGCTACAACCAGCCTGGAGAGATTTGTATCCGAGGCCAACAGATTATGAAAG<br>GATACCTTAACGACGAGGCGGCTACCGCTTCCACTATTGACTCGGAGGGTTGGTTGCACACTGGCGATGTGGGTTACGTGGAC<br>GATGACGACGAAATCTTCATCGTCGACCGAGTGAAGGAGCTCATTAAGTACAAGGGCTTTCAAGTGCCCCCTGCTGAACTGGA<br>GGGGCTGCTTGTGTCACACCCCTCGATTGCCGACGCTGCCGTCGTCCCTCAGAAAGACGTGGCCGCTGGAGAAGTCCCCGTGG<br>CCTTCGTCGTAAGGTCCAACGGCTTCGATCTTACCGAGGAGGCCGTCAAGGAATTTATTGCCAAACAGGTCGTCTTCTATAAG<br>CGATTGCATAAGGTTTATTTCGTCCACGCAATTCCTAAATCCCCATCAGGAAAAATATTAAGAAAAGATCTC<br>CGCGCCAAACTAGAAACCGCCGCCACTCAGACCCCCTAA
AdC2′H	ATGGCACCATCCACAACCGACGATTATAGACATTTTGCAGTAACCAAGGGACACGGAGTTAAAGGATTATCTGACTTACAATT<br>AGGAGCATTACCAGAACAGTACATACAACCAGTAGAAGAGCGACTCGATATGACCAAAGTCCTCAAAGAGGAGTCTATTCCCG<br>TGATCGACATGTCGAATCTTGACGACCCCAAGGTGGCTGAGCAAATCGCTGCTGCCGCCGAGAAGTGGGGGTTCTTCCAGATT<br>GTGAACCACGGTGTGCCCATCGAGGTTCTGGAAAACGTGCAGGAGGCGACACGTCGATTTTTCGCGCTGCCTGTGGAGGAGAA<br>GATCAAGTTCACCCAGGAACATAGTCCAACAAACTCAGTTCGCCTCACCACCTCCTTTCTTCCCAAAATTGATAAGGTCCTTG<br>AATGGAAGGATTACCTGTCGATTTTGGTCTCCGACAAGAAGTCTT<br>CCGAATTCTGGCCTTCTGCCTGTAAGCAGGAGGTCATGGAATACGTGGAAAAGTCCGAGTTCGTGATGAAGTGGTTGCTCAAG<br>GCTCTCATGCAAGGTCTTAACGTCGACATGGATAGCAAGGAGTCTCTGCTGATGGGCTCTACCCGAATCAACCTCAACTATTA<br>CCCTGTTTGTCCGAACCCTGAGCTGGCCATTGGTGTGGGCAGACACTCGGATGTTAGCTCTCTCACTTTTCTGCTCCAGGACA<br>ATGTGGGAGGTCTGCACATCCGGAAAATGGAGACTGATACGTGGATCTACGTCCCTCCCATTACTGGAGCCATCGTAATCAAC<br>ATTGGAGACGCTCTGCAGATCCTATCGAATGGCAAGTACAAGTCGGCCGAACACTGCGTCGCAGCCAACGGCTCCCATAACCG<br>AATTTCAGTTCCTATTTTTGCTAACCCCAGCCCAGACGACATAATTGGCCCGCTGCCCGAGGTGCTCAAGAACGGAGAGAAGC<br>CCATTTATAAACATGTACTATACTCAGACTACGTAAAACACTTCTACAGAAAATCTCACGACGGAAAAGACACATTAGATTTC<br>GCCAAAATGTAA
RgC2′H	ATGGCACCAACAAAAGACTCAGTAATACATATGGGGGCAGAATCATGGGATGAAATAAGCGAATTTGTAACGAAAAAAGGACA<br>CGGAGTTAAAGGATTATCTGAACTAGGCATTAAAACACTACCAAAACAATTTCATCAACCTCTGGAGGAAAGATTCTCCGAAA<br>AGAAAATTCTGGAGCGTGCTAGCATTCCATTGATCGACATGTCGAAGTGGGACTCTCCTGAGGTGGTGAAGTCGATCTGCGAC

	GCCGCGGAGCACTGGGGGTTCTTCCAGATTGTGAACCACGGTGTGCCCTTGGAGACCCTTCAGAGGGTCAAGGAGGCCACTCA<br>CCGATTTTTCGCGCTGCCTGCCGAAGAGAAGAACAAGTACTCTAAGGAGAACTCGCCTATCAATAACGTACGATTCGGGTCGT<br>CGTTTGTTCCTCATGTGGAGAAAGCACTTGAATGGAAGGATTTTTTATCAATGTTTTACGTGTCCGAGGAGGAGACAAACACC<br>TACTGGCCTCCGATCTGTCGAGACGAGATGCTCGAGTACATGCGATCATCTGAAGTTCTCATTAAGCGTTTGATGGAGGTTCT<br>GGTTGTCAAGGGTCTGAAGGTGAAACAGATCGATGAGATTAGAGAACCTATGCTGGTGGGCTCTCGGCGAATCAACCTCAACT<br>ATTACCCTAAATGTCCGAACCCTGAGCTGACGCTTGGTGTGGGCAGACACTCGGATATCAGCACTTTCACCATTCTGCTCCAG<br>GACGAGATCGGAGGTCTGCACGTTCGCAAGCTAGATGACACGGGTAACACTTGGGTCCACGTGACACCCATCTCCGGCTCCCT<br>CATCATCAACATTGGAGACGCTCTGCAGATCATGTCAAATGGACGATATAAGTCCATCGAACATATGGTCGTCGCCAACGGAA<br>CCCAGGATCGAATTTCAGTTCCTCTGTTTGTGAACCCCAAGCCCCAGGCAATTCTCTGTCCCTTCCCCGAGGTGCTCGCCAAC<br>GGAGAGAAGCCCGTATACAAGCCAGTACTATGCTCAGATTACTCCCGACACTTCTACACTAAACCCCACGACGGAAAAAAAAC<br>AGTTGACTTTGCTCTCATGAACTAA

Supplementary Table S2. Plasmids used and constructed in this study

Plasmids	Description	Source
pUC-Kan-tCYC1-pUAS1B8TEF-pTEFin-tXPR2	For the synthesis of the tXPR2-pTEFin-pUAS1B8TEF-tCYC1 expression cassette; Kanamycin-resistant (Kan+)	Synthesized by Genewiz Biotechnology Co., Ltd. (Suzhou, China)
pHR_AXP_hrGFP	Episomal expression vector with URA3 screening marker; Ampicillin-resistant (Amp+)	Ref. 21
p-CASyl	Cas9-sgRNA gene-editing vector with LEU2 screening marker; Ampicillin-resistant (Amp+)	Ref. 22
pHRm-URA3	Episomal expression vector carrying the tXPR2-pTEFin-pUAS1B8TEF-tCYC1 expression cassette, with URA3 as a screening marker	This study
pYarrow-LEU2	Episomal expression vector carrying the tXPR2-pTEFin-pUAS1B8TEF-tCYC1 expression cassette, with LEU2 as a screening marker	This study
pHRm-MFE1	Integrative expression vector carrying MFE1 homologous arms	This study
p-CASyl-MFE1	CRISPR-Cas9 gene-editing vector with sgRNA targeting the MFE1 locus	This study
pHRm-(MFE1)-AtC4H-AtCPR	Site-specific integrative vector targeting the MFE1 locus for expression of *AtC4H* and *AtCPR*	This study

pYarrow-FjTAL	Episomal expression vector for expression of *FjTAL*	This study
pYarrow-ClTAL	Episomal expression vector for expression of *ClTAL*	This study
pYarrow-EcaroL	Episomal expression vector for expression of *EcaroL*	This study
pHRm-AtPAL	Episomal expression vector for expression of *AtPAL*	This study
pHRm-SmPAL	Episomal expression vector for expression of *SmPAL*	This study
pHRm-RtPAL-TAL	Episomal expression vector for expression of *RtPAL-TAL*	This study
pHRm-(MFE1)-AtC4H-AtCPR-RtPAL-TAL	Site-specific integrative vector targeting the MFE1 locus for co-expression of *AtC4H*, *AtCPR* and *RtPAL-TAL*	This study
pHRm-ScARO4^{K229L}-ScAR07^{G141S}	Episomal expression vector for co-expression of *ScARO4*^{K229L} and *ScARO7*^{G141S}	This study
pHRm-YlARO4^{K221L}-YlAR07^{G139S}	Episomal expression vector for co-expression of *YlARO4*^{K221L} and *YlARO7*^{G139S}	This study
pHRm-(MFE1)-AtC4H-AtCPR-RtPAL-TAL-ScARO4^{K229L}-ScAR07^{G141S}-EcaroL	Site-specific integrative vector targeting the MFE1 locus for co-expression of *AtC4H*, *AtCPR*, *RtPAL-TAL*, *ScARO4*^{K229L}, *ScARO7*^{G141S} and *EcaroL*	This study
pHRm-D17	Integrative expression vector carrying D17 homologous arms	This study
p-CASyl-D17	CRISPR-Cas9 gene-editing vector with sgRNA targeting the D17 locus	This study
pHRm-(D17)-RgC2'H	Site-specific integrative vector targeting the D17 locus for *RgC2′H* gene expression	This study

pHRm-(D17)-AdC2'H	Site-specific integrative vector targeting the D17 locus for *AdC2′H* gene expression	This study
pHRm-(D17)-AtCOSY	Site-specific integrative vector targeting the D17 locus for *AtCOSY* gene expression	This study
pYarrow-At4CL3	Episomal expression vector for *At4CL3* gene expression	This study
pYarrow-Sc4CL	Episomal expression vector for *Sc4CL* gene expression	This study
pYarrow- Gm4CL4	Episomal expression vector for *Gm4CL4* gene expression	This study
pHRm-(D17)-AtCOSY-RgC2'H-At4CL3	Site-specific integrative vector targeting the D17 locus for expression of *AtCOSY*, *RgC2′H* and *At4CL3*.	This study

Supplementary Table S3. Primers used in this study

**Primer**	**Sequence (5′–3′)**	**Used for**
HR-F	CGTACGGTACCGCATGCTTCCTTGGT	Construction of the ‘pHRm-URA3’ vector
HR-R	TCCGGATAAGCTTGGCGTAATCATGGT	
phr0-F	CCAAGGAAGCATGCGGTACACTAGTCTGTCATGAGATAAGCTGTC	
phr0-R	CCATGATTACGCCAAGCTTAACTAGTCAGCTGCCATGGAAAACCT	
pY-F	TCATGACAGCTGACGCGTGATCCCCCTT	Construction of the ‘pYarrow-LEU2’ vector
pY-R	CCATGGCAGCTGGTTTAAACAAAC	
pY0-F	GATCACGCGTCAGCTGTCATGAGATAAGCTGTCAAACATGAGAATT	
pY0-R	TGTTTAAACCAGCTGCCATGGAAAACCTTCTCAAGCAAGGTTTTC	
sgRNA-R	CAACCCGGTCTCTGTCGACAAA	Construction of the ‘p-CASyl-MFE1’ vector
sgRNA-F	CAAGCTTGTTTGTTTAAACAGAGACCG	
sgMFE1-F	TCTAAAACAGTCTCACCGAAACCAACCAGACGAGCTTACTCGTTTCGT	
sgMFE1-R	CGTCTGGTTGGTTTCGGTGAGACTGTTTTAGAGCTAGAAATAGCA	

MFE1-F1	ACCAAGGAAGCATGCGGTACAGCATCTCCTCAGTGAAGAC	Amplification of MFE1 homologous arm
MFE1-R1	ACAGCTTATCTCATGACAGCTGGTTTAAACTTGGTTTCGGTGAGACTC	
MFE1-F2	TGAGAAGGTTTTCCATGGCAGCTGATGCATCCAGGGCGAGCTTGGCA	
MFE1-R2	CCATGATTACGCCAAGCTTAACAAGCTTCTGTATGGATGTG	
phrMFE1-F	GAGTCTCACCGAAACCAAGTTTAAACCAGCTGTCATGAGATAAGCTGT	Construction of the 'pHRm-MFE1' vector
phrMFE1-R	TGCCAAGCTCGCCCTGGATGCATCAGCTGCCATGGAAAACCTTCTCA	
RT-F	GAGAAGGTTTTAGTAGGATGAAACGATAAGCTGTCAAACATGAGA	Amplification of *RtPAL-TAL* gene
RT-R	CTCGCCCTGGAATGCATCACNNNGTGAGAGACCGGGTTGGCGGCGT	
Sc4-F	CAAGACCGGCAACGTGGGGTTAATTAACTATTTCTTGTTAACTTCTC	Amplification of *ScARO4* and *ScARO7*
Sc4-R	GCAGTACTAACCGCAGCCTAGGATGAGTGAATCTCCAATGTTC	
Sc7-F	AAGAATCATTCAAAGGCGCGCCATGGATTTCACAAAACCAGA	
Sc7-R	GATCTTAGCTAGCCGCGCGATCGCTTACTCTTCCAACCTTCTTA	
Yl4-F	ACCGGCAACGTGGGGTTAATTAATTAGTTCTTGTTTCGTCGCT	Amplification of *YlARO4* gene
Yl4-R	TGCAGTACTAACCGCAGCCTAGGATGTCCCGTTCCTCCTCTCC	
Yl7-F	AGAATCATTCAAAGGCGCGCCATGGACTTCACTAAAGCCGA	Amplification of *YlARO7* gene
Yl7-R	ATCTTAGCTAGCCGCGCGATCGCCTACTCCAACCGCCGGAGCA	
2Sc1-F	TGATAGCAGCAACACCATGCAGAGTAACACC	Construction of the 'pHRm-ScARO4^{K229L}-ScARO7^{G141S}' vector
2Sc1-R	TCTCTAGTGGCAACAGAAGAGAAGTTATTC	
2Sc2-F	GATAAGAATAACTTCTCTTCTGTTGCCACTA	
2Sc2-R	TCATGGGTGTTACTCTGCATGGTGTTGCTGC	
2Yl1-F	ATGGCGGCAACACCCTGCAGGGTGACACCCA	Construction of the 'pHRm-YlARO4^{K221L}-YlARO7^{G139S}' vector
2Yl1-R	ACACCATGACCGAAGAATAGTTCTCGGGCTG	
2Yl2-F	TCAGCCCGAGAACTATTCTTCGGTCATGGTG	
2Yl2-R	ACTTCATGGGTGTCACCCTGCAGGGTGTTGC	
3Yl-F	GAGTCTCACCGAAACCAAGTTTAAACCAGCTGTCATGAGATAAGCTGT	Construction of the 'pHRm-(MFE1)-AtC4H-AtCPR-RtPAL-TAL-
3Yl-R	GACAGCTTATCTGCATCAGCTGCCATGGAAAACCTTCTCAAGCAAGGT	
EL-F	GAAGGTTTTCCATGGCAGCTGATGCAGATAAGCTGTCAAACATGAGA	
EL-R	GCTTATCTCGGCTGGGTTAGTGGCATAGAGACCGGGTTGGCGGCGTA	

		ScARO4^{K229L}-ScARO7^{G141S}-EcaroL' vector
sgD17-F	ATTTCTAGCTCTAAAACTCCGTAATATAGGTGACGACGACGAG	Construction of the 'p-CASyl-D17' vector
sgD17-R	ATTTCTAGCTCTAAAACTCCGTAATATAGGTGACGACGACGAG	
sgRNA-R	CAACCCGGTCTCTGTCGACAAA	
sgRNA-F	CAAGCTTGTTTGTTTAAACAGAGACCG	
HR-F	CGTACGGTACCGCATGCTTCCTTGGT	Construction of the 'pHRm-D17' vector
HR-R	TCCGGATAAGCTTGGCGTAATCATGGT	
D17-F1	GAAGCATGCGGTACCGTACGTCTGTACTGGACAGATTTTG	Amplification of D17 homologous arm
D17-R1	AGCTTATCTCATGACAGCTGGTTTAAACGTCACCTATATTACGGAAGC	
D17-F2	AAGGTTTTCCATGGCAGCTGATGCATGACGGGAGATAGAAGACTAG	
D17-R2	GATTACGCCAAGCTTATCCGGAGTCTCTTCAACTCGCTCAAA	
phrD17-F	GTGACGTTTAAACCAGCTGTCATGAGATAAGCTGTCAAACATGAG	Construction of the 'pHRm-D17' vector
phrD17-R	CCGTCATGCATCAGCTGCCATGGAAAACCTTCTCAAGCAAGGT	
Rg-F	GACCGGCAACGTGGGGTTAATTAATTAGTTCATGAGAGCAAAGT	Construction of the 'pHRm-(D17)-AtCOSY-RgC2'H-At4CL3 vector
Rg-R	TGCAGTACTAACCGCAGCCTAGGATGGCACCAACAAAAGACTC	
At-F	GTTTTCCATGGCAGCTGATGCAGCTAGCCTCGAGTAGTGGTACC	
At-R	CTAGTCTTCTATCTCCCGTCAATGCATCAGCTGCCATGGAAAACCTTCTCAA	

Supplementary Table S4. Strains constructed in this study

**Strains**	**Genotype**	**Sources**
Po1f	MATa, Ura3-302, Leu2-270, XPR2-322, axp2-deltaNU49, XPR2:: SUC2	ATCC (MYA-2613)
SC52+SC59	Polf, AXP::ScRAD52+ScRAD59,ΔKu70	Ref. 20
YL-P	SC52+SC59, MFE1::AtCPR+AtC4H+EcaroL+RtPAL-TAL+ScAR07^{G141S}+ScARO4^{K229L}	This study
YL-PU	YL-P, D17:: RgC2′H+At4CL3+AtCOSY	This study

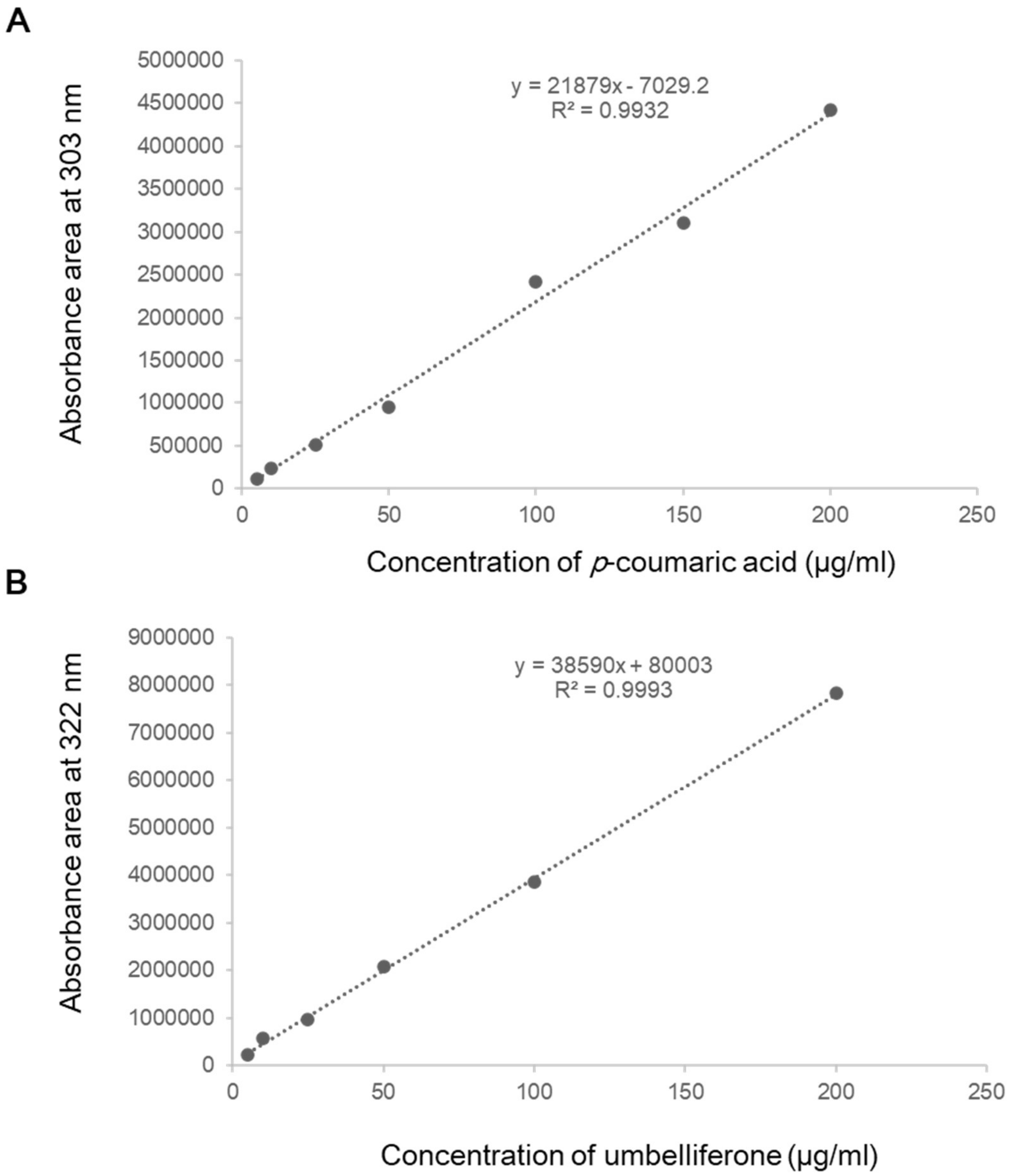

Supplementary Figure S1. Calibration curves of *p*-coumaric acid and umbelliferone standards. (A) Linear regression curve for *p*-coumaric acid detected at 303 nm; (B) Linear regression curve for umbelliferone detected at 322 nm. The injection volume was 10 µL. Peak area was plotted against analyte concentration (µg/mL).

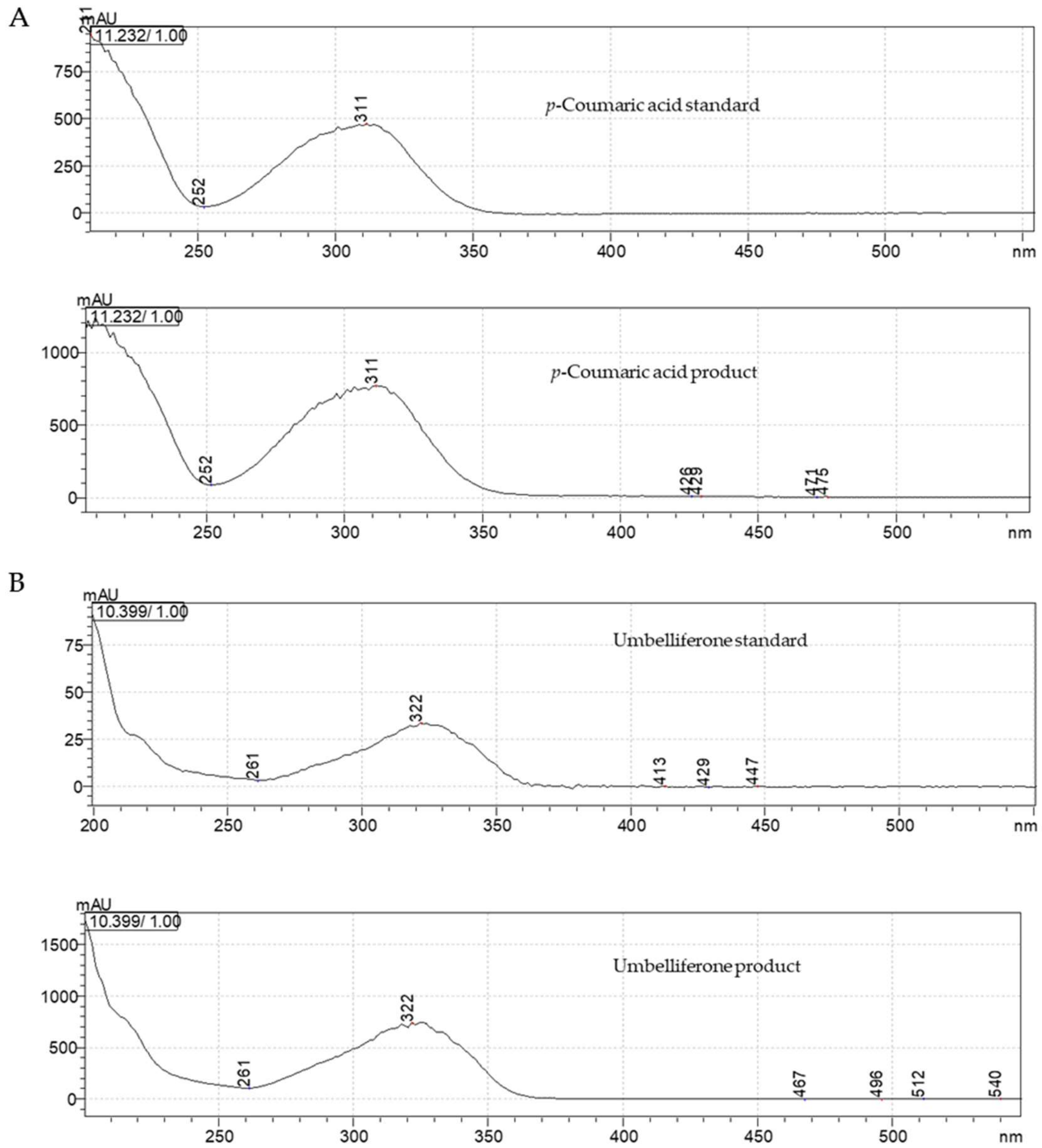

Supplementary Figure S2. UV absorption spectra of *p*-coumaric acid (A) and umbelliferone (B) products in comparison with their corresponding standards. The spectra were acquired with a DAD detector during HPLC analysis.

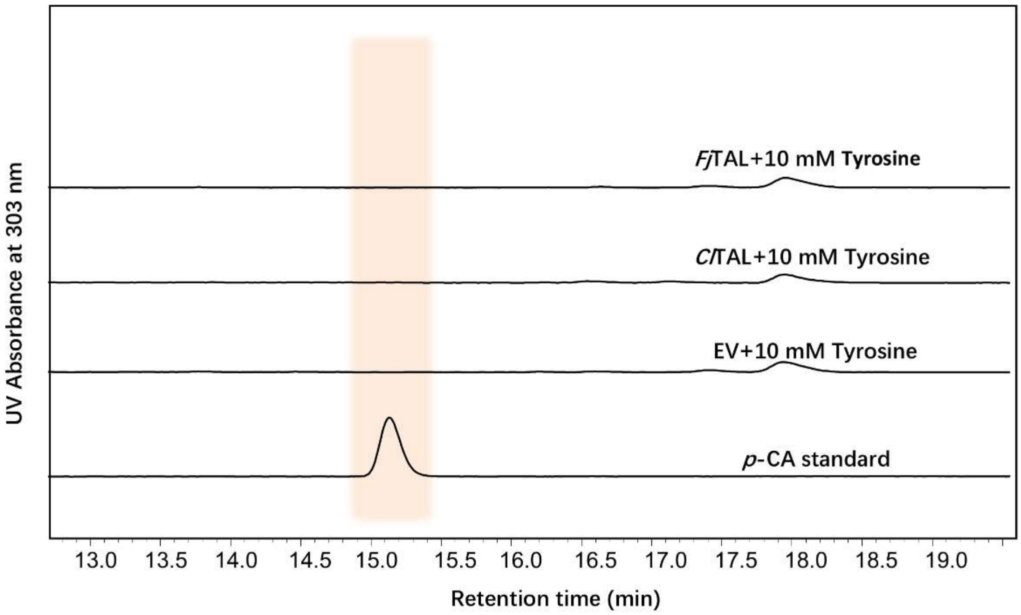

Supplementary Figure S3. HPLC analysis of culture supernatants from strains expressing FjTAL, ClTAL, and empty-vector control (EV) fed with 10 mM tyrosine. The chromatogram of *p*-CA standard is shown for peak reference. No obvious *p*-CA peak was observed in all three fermentation samples compared with the authentic *p*-CA standard.

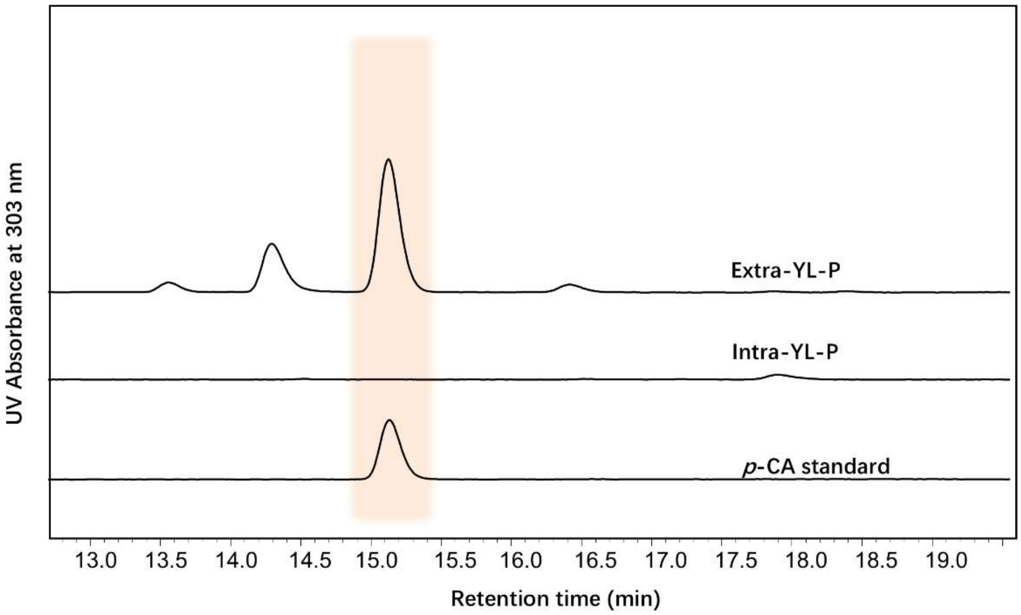

Supplementary Figure S4. HPLC-UV chromatograms monitored at 303 nm showing extracellular versus intracellular *p*-coumaric acid (p-CA) formation in engineered yeast YL-P. Extra-YL-P: secreted extracellular fraction; Intra-YL-P: intracellular fraction; *p*-CA standard: authentic *p*-CA standard. The characteristic *p*-CA peak was prominent in the extracellular sample and nearly undetectable intracellularly.

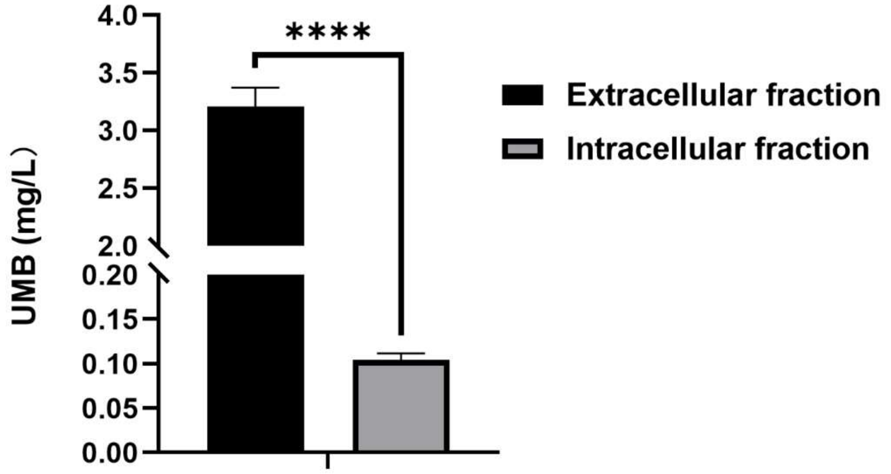

Supplementary Figure S5. Comparison of extracellular and intracellular umbelliferone (UMB) titers in engineered yeast strain YL-PU under shake-flask fermentation. Data are presented as mean ± standard deviation (SD), n = 3 biological replicates. Statistical significance was defined as *P*< 0.05. Significance level: ****, *P* < 0.0001.
